# Dynamic Regulation of Tgf-B Signaling by Tif1γ: A Computational Approach

**DOI:** 10.1371/journal.pone.0033761

**Published:** 2012-03-23

**Authors:** Geoffroy Andrieux, Laurent Fattet, Michel Le Borgne, Ruth Rimokh, Nathalie Théret

**Affiliations:** 1 Inserm U1085-IRSET, Université de Rennes 1, Rennes, France; 2 Université de Rennes 1, IRISA, Rennes, France; 3 Inserm U1052/CNRS 5286, Centre de Recherche en Cancérologie de Lyon, Lyon, France; Indian Institute of Science, India

## Abstract

TIF1γ (Transcriptional Intermediary Factor 1 γ) has been implicated in Smad-dependent signaling by Transforming Growth Factor beta (TGF-β). Paradoxically, TIF1γ functions both as a transcriptional repressor or as an alternative transcription factor that promotes TGF-β signaling. Using ordinary differential-equation models, we have investigated the effect of TIF1γ on the dynamics of TGF-β signaling. An integrative model that includes the formation of transient TIF1γ-Smad2-Smad4 ternary complexes is the only one that can account for TGF-β signaling compatible with the different observations reported for TIF1γ. In addition, our model predicts that varying TIF1γ/Smad4 ratios play a critical role in the modulation of the transcriptional signal induced by TGF-β, especially for short stimulation times that mediate higher threshold responses. Chromatin immunoprecipitation analyses and quantification of the expression of TGF-β target genes as a function TIF1γ/Smad4 ratios fully validate this hypothesis. Our integrative model, which successfully unifies the seemingly opposite roles of TIF1γ, also reveals how changing TIF1γ/Smad4 ratios affect the cellular response to stimulation by TGF-β, accounting for a highly graded determination of cell fate.

## Introduction

Complex signaling by transforming growth factor β(TGF-β) forms a pivotal network that plays an essential role in tissue homeostasis and morphogenesis. At the same time, up-regulation and activity of TGF-β has been linked to various diseases, including fibrosis and cancer, by promoting cell proliferation and invasion and the epithelial-mesenchymal transition [1]. TGF-β signaling occurs through association with a heteromeric complex of two types of transmembrane serine/threonine kinases, the type I (TβRI) and type II (TβRII) receptors. TGF-β binding to TβRII induces recruitment and phosphorylation of TβRI, which in turn transmits the signal through phosphorylation of the receptor-bound R-Smad transcription factors, Smad2 or Smad3. Once phosphorylated, the R-Smads hetero-dimerize with their common partner, Smad4. The resulting complexes then migrate to the nucleus, where they regulate the transcription of TGF-β-target genes in conjunction with other transcription factors [2].

Nuclear Transcriptional Intermediary Factor 1 γ, TIF1γ (also known as tripartite motif protein TRIM33), is a member of the transcriptional intermediary factor 1 family [3] and was recently identified as a new partner of the Smad-dependent TGF-β signaling pathway. A screen for molecules involved in the specification of the embryonic endoderm first revealed TIF1γ as a Smad4-binding protein and as a negative regulator of TGF-β signaling [4]. TIF1γ mono-ubiquitinates Smad4, inducing its nuclear export to the cytoplasm, where the FAM/UPS9x deubiquitinating enzyme was recently shown to allow Smad4 recycling [5]. The role of TIF1γ as a repressor was also reported in the control of Smad activity during embryogenesis [6]. In contrast, TIF1γ was identified as a protein partner for receptor-activated Smad2/3, resulting in an alternative positive regulatory Smad4-independent TGF-β signaling pathway [7].

Whether TIF1γ down-regulates or promotes alternative TGF-β signaling may be linked to the cellular context. TIF1γ is a ubiquitous protein and its mRNA has been detected in all tissues [8]. Its loss of expression has been shown to favor Kras^G12D^-dependent precancerous pancreatic lesions [9], induce cell-autonomous myeloproliferative disorders in mice [10] and potentiate TIF1α-induced murine hepatocellular carcinoma [11], thereby supporting a protective role of TIF1γ in cancer. Consistent with this view, a decrease in TIF1γ expression in human pancreatic cancer and human chronic myelomonocytic leukemia has been reported [9], [11] and TIF1γ silencing in human mammary epithelial cell lines was shown to lead to a strong epithelial-mesenchymal transition mediated by TGF-β1 [12]. In contrast, a pro-tumorigenesis role for TIF1γ has been suggested by the observation that its expression prevents Smad4-mediated growth inhibition in response to TGF-β [4]. In line with the uncertain role of TGF-β in cancer, TIF1γ may differentially affect TGF-β signaling according to the cellular context by acting either as tumor suppressor or promoter.

Several mathematical models have been developed to predict the dynamic behavior of TGF-β signaling. In particular, initial differential models that couple signaling with receptor trafficking have significantly improved our understanding of the plasticity of the TGF-β signaling pathway [13]. Models focusing on Smad phosphorylation [14], Smad nucleocytoplasmic shuttling [15], [16] and Smad oligodimerization [17] have also been developed to understand the dynamics and flexibility of Smad-dependent pathways, while integrative models have coupled receptor trafficking to Smad pathways [18]–[20]. As the latter models recapitulate the essential components of the canonical Smad-dependent TGF-β signaling pathway, they constitute useful tools to investigate the role of new regulatory components of TGF-β signaling.

We have used an integrative modeling approach to explore the impact of TIF1γ on the outcome of TGF-β signaling. Taking advantage of mathematical models of receptor trafficking [13] and Smad shuttling [16], we have developed a new TGF-β signaling model that includes TIF1γ and FAM/UPS9x. Our model, which is based on the transient formation of a ternary complex containing TIF1γ Smad4 and Smad2/3, successfully reconciles the different observations reported for TIF1γ-Smad4 [4] and TIF1γ-Smad2/3 [7] interactions. We show that TGF-β signaling is highly sensitive to the TIF1γ/Smad4 ratio, suggesting a critical role for the FAM/UPS9x deubiquitinase. This model also predicts how varying TIF1γ/Smad4 ratios can modulate the cellular response to transient and sustained TGF-β stimulation, accounting for a highly graded TGF-β response. We discuss how the seemingly opposite roles of TIF1γ may be resolved by taking into account the dynamic balance of interactions involving Smad4 and Smad2/3.

## Materials and Methods

### Mathematical modeling

The model consists of a system of nonlinear, ordinary differential equations that merge the ODE models of receptor trafficking [13] and Smad shuttling [16]. Briefly, the receptors described in the Smad shuttling model were replaced by those of the receptor trafficking model using unit conversion in a cell volume of 2.27×10^−12^L. Model building, parameters, system ordinary equations and description of the model in Systems Biology Markup Language (SBML) are detailed in Tables S1 and S2 and Model S1. Model simulations were implemented with the mathematical Scipy library of Python language programming and the Matplotlib Python 2D plotting library was used to visualize the simulation curves.

### Cell culture and siRNA transfection

Human mammary epithelial (HMEC) cells infected with a retrovirus carrying hTERT and the oncogenic H-RasV12 (HMEC-TR) allele were provided by R. A. Weinberg [21] and cultured as previously described [12]. Cells were transfected with 5 nM siRNA and 0.5 µl/ml lipofectamine RNAiMax (Invitrogen) and further cultured in the presence or absence of 10 ng/ml TGF-β1 (Peprotech) for the indicated times.

### Chromatin immunoprecipitation (ChIP)

Assays were carried out on cells transfected with the PAI-1 p800-Luc construct, as previously described [12], using the kit from Upstate Biotechnology. Briefly, cell lysates were subjected to anti-Smad4 (SantaCruz) or anti-TIF1γ (Bethyl) immunoprecipitation. Smad4- or TIF1γ-precipitated genomic DNA was subjected to PCR. The 351-bp PAI-1 promoter region harboring the Smad-binding elements was amplified with primers 5′-AGCCAGACAAGGTTGTTG-3′ and 5′-GACCACCTCCAGGAAAG-3′. An unrelated genomic DNA sequence (actin) was amplified with primers 5′-AGCCATGTACGTTGCTATCCAG-3′ and 5′-CTTCTCCTTAATGTCACGCACG-3′.

### Relative quantification of mRNA by real-time PCR

Real-time quantitative PCR was performed using the qPCRTM Core Kit for SybrTM Green I from Eurogentec and the ABI Prism 7700 thermocycler (Perkin-Elmer, Foster city, CA, USA). Primer pairs for target genes were: sense CDH11 (OB-Cadherin), 5′CCC TGA AAT CAT TCA CAA TCC3′, antisense 5′AGT CCT GCT TCT GCC GAC T3′; CDH2 (N-Cadherin), sense: 5′GTG CAT GAA GGA CAG CCT CT3′, antisense: 5′ATG CCA TCT TCA TCC ACC TT3′; HPRT, sense: 5′TGA CCT TGA TTT ATT TTG CAT ACC3′, antisense: 5′CGA GCA AGA CGT TCA GTC CT3′.

### Western blot analysis

Cell lysates were subjected to SDS-polyacrylamide gel electrophoresis and transferred onto PVDF membranes. The blots were incubated for 1 hr in Tris-buffered saline containing 0.1% Tween 20 and 5% non-fat dry milk and further incubated for 1 hr with specific primary antibodies (anti-Smad4, SantaCruz biotechnology; anti-TIF1γ, Euromedex). The bound antibodies were visualized with horseradish peroxidase–conjugated antibodies using the ECL-Plus reagent (Roche).

## Results and Discussion

### Quantitative models for TIF1γ-dependent TGF-β signaling

**Figure 1:**
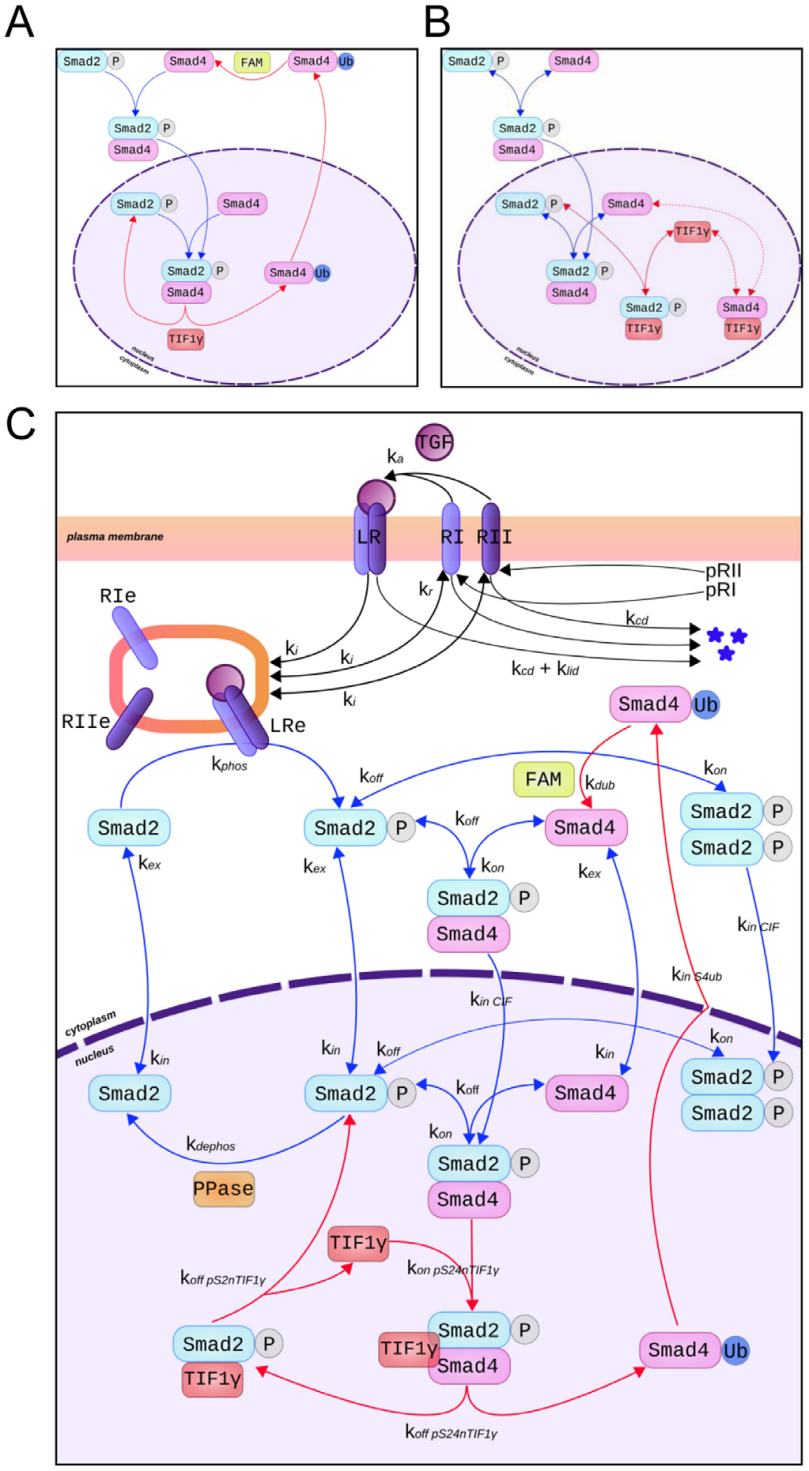
Schematic representation of the models. Detailed information on parameters and entities are given in Tables S1 and S2. A) Model hypothesis from [4]. B) Model hypothesis from [7]. C) Integrated model including TIF1γ (red rectangle) and FAM (green rectangle).

Merging receptor trafficking [13] and Smad cytonucleoplasmic shuttling [16] models through their common receptor-ligand complex in the endosome (LRe), we developed new models that integrate TIF1γ. Kinetic parameters were estimated according to the experimental data from [5] and [7] and are detailed in Table S1. We first constructed two separate models, each taking into account the different hypotheses regarding Smad/TIF1γ interactions. The first model is based on the TIF1γ-dependent negative regulation associated with the ubiquitination of Smad4 ([4], [5]; Figure 1A). In this model, TIF1γ interacts preferentially with Smad4 within phosphorylated Smad2-Smad4 complexes in response to TGF-β, leading to a rapid dissociation of complexes and formation of ubiquitined Smad4 (Smad4ub) that is exported from the nucleus. Similar to the transient interaction of the phosphatase (PPase) with phosphorylated Smad2 [15], [16], the formation of TIF1γ-Smad complexes was neglected because of fast reaction rates. In the cytoplasm, ubiquitinated Smad4 undergoes deubiquitination by FAM/UPS9x (FAM), thereby recycling Smad4 for TGF-β signaling (Figure 1A). We set the same kinetic parameters for association between TIF1γ and phosphorylated Smad2-Smad4 complexes in the nucleus (pS2S4n) and association between phosphorylated Smad2 and Smad4. Ubiquitination/deubiquitination and phosphorylation/dephosphorylation kinetics were considered to be similar, as previously described [5]. Export of ubiquitinated Smad4 from the nucleus to the cytoplasm was assumed to be 2-fold higher than entry of Smad4 in the nucleus, based on the observation suggesting that ubiquitined Smad4 is less efficiently retained in the nucleus [4], [5].

Our second model is based on results from He *et al.* [7], who proposed that TGF-β induces a competing interaction between TIF1γ and phosphorylated Smad2, although an association of TIF1γ with Smad4 was also detected in the nucleus (Figure 1B). In the absence of conclusive experimental data, we considered the kinetic parameters for association between TIF1γ and either phosphorylated Smad2 or Smad4 in the nucleus to be similar to those for phosphorylated Smad2 with Smad4. To test this hypothesis, we analyzed the effect of a 2-fold decrease in k_on_/k_off_ for the association between TIF1γ and Smad2 or Smad4, which did not modify the TGF-β response in simulation studies.

Finally, we integrated the TIF1γ and FAM/UPS9x modulators into a unique model that merges all experimental observations (Figure 1C). Unlike the model depicted in Figure 1A, we considered TIF1γ binding to Smad4 as part of a ternary complex, in which phosphorylated Smad2, Smad4 and TIF1γ are associated in the nucleus (pS24nTIF1γ). In this case, note that the interaction of TIF1γ with Smad2 occurs within phosphorylated Smad2-TIF1γ (pS2nTIF1γ) complexes that are generated by dissociation of the ternary complexes in the nucleus. We set the same kinetic parameters for the formation/dissociation of the ternary pS24nTIF1γ complexes and the formation/dissociation of the phosphorylated Smad2-Smad4 complexes.

### Model analysis and simulation

**Figure 2:**
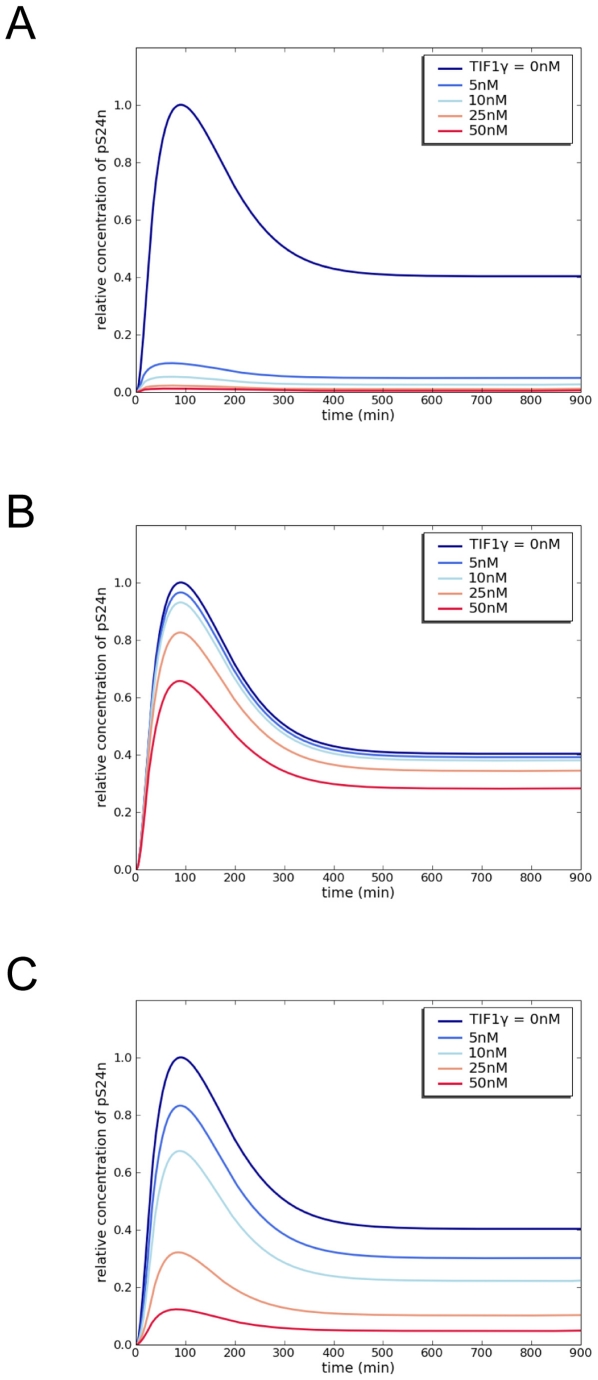
Effect of TIF1γ on TGF-β signaling. Modeling analysis of the pS24n response to increasing TIF1γ concentrations at a 10 nM TGF-β input. A) Model according to [4]; B) model according to [7] and C) integrated model.

We next performed computational experiments to investigate the dynamics of TGF-β signaling according to each model. TGF-β signaling was expressed as the amount of phosphorylated Smad2-Smad4 complexes in the nucleus (pS24n) because TGF-β target genes are regulated by these heterodimeric complexes. To explore the functional effect of TIF1γ on the TGF-β transcriptional signal, simulation studies were performed using different concentrations of TIF1γ varying from 0 to 50 nM, the latter corresponding to the initial concentration of Smad4 (Figure 2, Table S1). These prediction studies showed that each model was either too sensitive, with total inhibition of signaling at low concentrations of TIF1γ according to the first model (Figure 2A), or too insensitive, with only a slight variation of signaling at higher TIF1γ concentrations according to the second model (Figure 2B). Each predictive model hence yielded a significant mismatch with the experimental data derived from the other. The strict negative regulatory role of TIF1γ proposed by Dupont *et al.* [4] is not compatible with the lack of sensitivity of the second model adapted from He *et al.* [7]. Similarly, He *et al.* observed a moderate TIF1γ effect on TGF-β transcriptional activity that did not agree with the high sensitivity of the first model adapted from Dupont *et al.* In contrast, our integrative model that includes all observations yielded a graded effect of TIF1γ on pS24n complex formation that is in agreement with the relative abundance of TIF1γ-Smad complexes reported in both studies, leading to a graded regulation of TGF-β signaling (Figure 2C).

**Figure 3:**
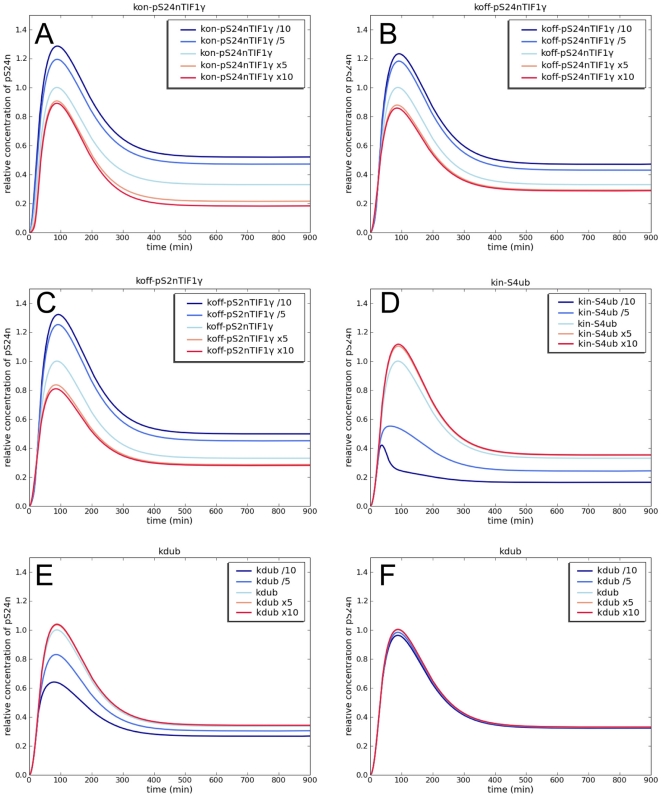
Parameter sensitivity analysis. Modeling analysis of pS24n response to variations of kinetic constants at a 10 nM TGF-β input. A) k_on_-pS24nTIF1γ, binding of TIF1γ to phosphorylated-Smad2/Smad4 complexes; B) k_off_-pS24nTIF1γ, dissociation of phosphorylated-Smad2/Smad4/TIF1γ complexes in the nucleus; C) k_off_-pS2nTIF1γ, dissociation of phosphorylated Smad2-TIF1γ complexes in the nucleus; D) k_in_-S4ub, nuclear export of ubiquitinated Smad4 in the cytoplasm; E) and F) k_dub_, deubiquitination of Smad4 according to relative FAM concentrations of 1 nM (E) and 10 nM (F).

To further explore the robustness of our integrative model, we evaluated the sensitivity of TGF-β signaling to variations in kinetic parameters. As shown in Figure 3, varying the rate of formation (Figure 3A) or dissociation (Figure 3B) of complexes containing TIF1γ and pS24n had little effect on TGF-β signaling. Similarly, varying the kinetic parameters for the dissociation of phosphorylated Smad2-TIF1γ complexes (pS2nTIF1γ) induced only few changes in the concentration of pS24n (Figure 3C). In contrast, TGF-β signaling was highly sensitive to the variation of k_in_-Smad4ub (Figure 3D), suggesting that the export rate of ubiquitinated Smad4 is a critical component of the regulation of TGF-β transcriptional activity. In addition, the slight alteration in TGF-β signaling induced by changes in the deubiquitination rate of Smad4 (Figure 3E) disappeared with increasing concentrations of the FAM deubiquitinase (Figure 3F), suggesting that changes in FAM expression might be a sensitive marker to predict modulation of TGF-β signaling. Taken together, the results of our simulation studies reveal a new pivotal role of the Smad4 ubiquitination/deubiquitination cycle in the regulation of the dynamics of TGF-β signaling. Of note is the predicted critical regulatory role of FAM in TGF-β signaling through Smad4 recycling.

### Experimental validation of the model

**Figure 4:**
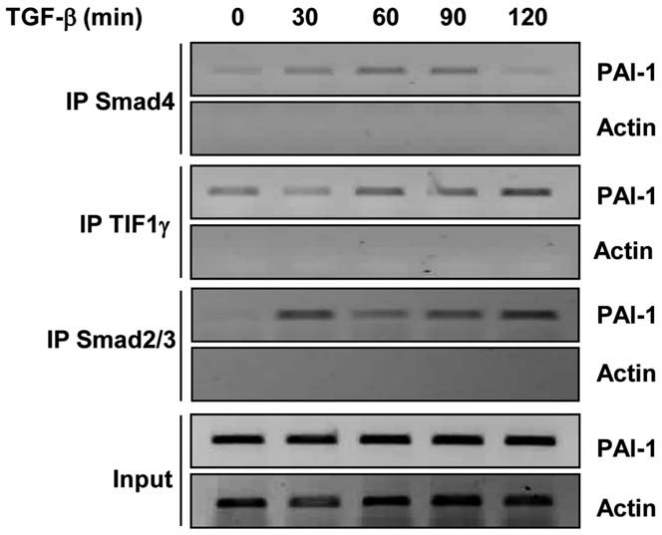
TIF1γ, Smad2 and Smad4 bind to the PAI-1 promoter. ChIP assays were performed on HMEC cells treated with TGF-β for the indicated times. Cell lysates were subjected to anti-Smad4 (IP Smad4), or anti-TIF1γ (IP TIF1γ), or anti-Smad2/3 (IP Smad2/3) chromatin immunoprecipitation. PCR amplification of the endogenous PAI-1 promoter (733/484) was performed to detect protein bound DNA. Primers specific to actin were used as controls.

A key component of our model is based on the hypothesis that a transient ternary complex is formed, associating Smad4, TIF1γ and Smad2. To investigate the reality of such an interaction, we performed chromatin immunoprecipation (ChIP) assays as previously described [12]. As shown in Figure 4, stimulation of cells with TGF-β induced the recruitment of Smad proteins on the promoter sequence of PAI-1, a TGF-β target gene. In the absence of TGF-β stimulation, TIF1γ showed a significant association with DNA while Smad2/3 was not detected. A faint Smad4 signal could be detected under these conditions. TGF-β stimulation led to the detection of a strong Smad2/3 ChIP signal. Between 30 and 90 min of TGF-β stimulation, the association of all three proteins with DNA appears consistent with the hypothesis that a ternary complex containing Smad4, Smad2/3 and TIF1γ transiently forms. After 120 min, Smad4 dissociated from DNA whereas Smad2/3 and TIF1γ remained present on the PAI-1 promoter. This observation is in agreement with our hypothesis that Smad2-TIF1γ complexes are released from the ternary complexes. Importantly, Dupont *et al.* [4], using a double-immunoprecipitation approach for TIF1γ and Smad4, previously reported formation of these ternary complexes. More recently TIF1γ was shown to be present at the promoter region of PAI-1 gene in uninduced cells, whereas an increase in TIF1γ association with the Smad-binding region of the promoter was also observed upon TGF-β stimulation [22].

**Figure 5:**
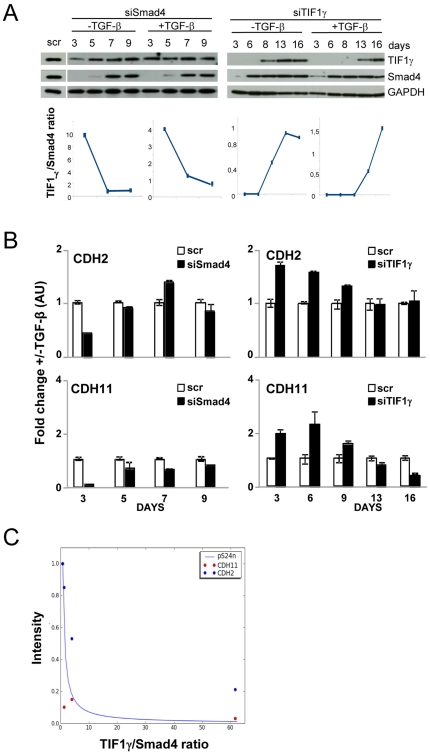
Expression of the CDH2 and CDH11 TGF-β target genes is sensitive to TIF1γ/Smad4 ratios. HMEC cells were transfected with Smad4 (siSmad4) or TIF1γ (siTIF1γ) siRNAs and cultured in the presence (+) or absence (−) of TGF-β for the indicated times (days). Controls were cells transfected with non-targeted siRNA (scr). A) Smad4 and TIF1γ protein levels were analyzed by immunoblotting (upper panels) and quantified by densitometric scanning (lower panels). B) TGF-β-induced fold changes in CDH2 and CDH11 expression were analyzed by RT–qPCR. All values were normalized to the amount of HPRT mRNA and expressed relative to the value obtained for TGF-β-untreated controls in arbitrary units (AU). Results are expressed as the mean ± SD of 3 independent experiments. C) mRNA levels of CDH2 (red circles) and CDH11(blue circles) were plotted against TIF1γ/Smad4 ratios and were fitted to the predictive equation curve of pS24n relative concentrations.

We next devised an experimental approach that could be used to evaluate TGF-β transcriptional activity as a function of variable TIF1γ/Smad4 ratios. Cells were transiently transfected with siRNAs to silence Smad4 or TIF1γ expression and were further stimulated or not with TGF-β for the indicated times (Figure 5A). The expression of Smad4 and TIF1γ was efficiently inhibited since no proteins were detected at day 3 post-transfection compared with cell transfected with non-targeted siRNAs (scr). The efficacy of RNA interference was confirmed at the mRNA level (Figure S1). This effect decreased with time according to siRNA availability and mRNA turnover, leading to the recovery of protein basal levels after several days (Figure 5A, upper panel). Note that silencing Smad4 and TIF1γ affected the amounts of TIF1γ and Smad4 proteins, respectively, detected at day 3. The time courses shown in Figure 5 finally allowed us to analyze cells containing variable amounts of endogenous Smad4 and TIF1γ proteins. For each time point, cell extracts were used for western blot analyses and TIF1γ/Smad ratios were evaluated by densitometric scanning of blots (Figure 5A, bottom panel). To perform this experimental verification, we quantified the mRNA levels of endogenous TGF-β target genes instead of using the over-expression of reporter genes to estimate transcriptional activities. We selected the CDH2 and CDH11 cadherin genes as they are up-regulated by TGF-β through Smad4- and TIF1γ-dependent pathways in our cell model (Figure S2). Using the same cell extracts used for western blotting (Figure 5A), the mRNA levels of CDH2 and CDH11 were quantified and TGF-β transcriptional activity was evaluated as the ratio of mRNA levels observed in the presence or absence of TGF-β (Figure 5B). TGF-β-induced expression of CDH2 and CDH11 was correlated with the amount of Smad4 and TIF1γ proteins. Compared to control cells (scr), low Smad4 expression (Day3) prevented TGF-β-dependent expression of CDH2 and CDH11 while the absence of TIF1γ led to up-regulation of CDH2 and CDH11.

We then compared these experimental data with results predicted by our integrative model. As shown in Figure 5C, our observations could be fitted to the simulation curves of TGF-β transcriptional signaling, a validation reinforced by the use of physiological parameters. We conclude from these results that TIF1γ is a new regulator that plays a pivotal role in the control of Smad4-dependent TGF-β transcriptional activity. These data also show that TIF1γ/Smad4 ratios can determine TGF-β-dependent transcriptional activity. Accordingly, our model supports the hypothesis of fast binding of TIF1γ to phosphorylated Smad2/Smad4 complexes and the release of both ubiquitinated Smad4 and phosphorylated Smad2-TIF1γ complexes.

### TGF-β dose- and time-dependent responses

**Figure 6:**
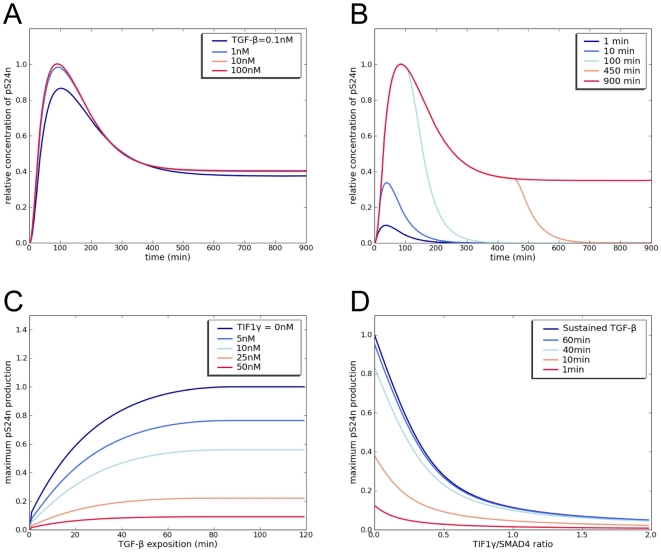
Concentration and time dependence of TGF-β signaling. A) and B) Modeling analysis of the pS24n response to increasing concentrations of TGF-β (A) and duration of stimulation with 10 nM TGF-β (B). C) and D) Modeling analysis of the maximum pS24n response as a function of TGF-β duration of exposure (C) or increasing TIF1γ/Smad4 ratios (D).

The concentration of TGF-β in the cellular microenvironment is highly variable and its increased expression has been reported in numerous pathologies, including inflammation, fibrosis and cancer [23]. However the determination of TGF-β concentrations at the cellular level within tissues remains a difficult task since TGF-β is stocked as a latent form in the extracellular matrix [24]. In addition, its conversion from latent to biologically active forms involves numerous protease- and non protease-dependent mechanisms that differ according to cell type and the physiological context, leading to a complex non-linear delivery [25]. All previous mathematical models are based on biological data obtained from *in vitro* experiments using either TGF-β concentrations (in the nM range) or on/off signal inputs. However, Zi and al. [19] recently developed an integrative model that includes a ligand depletion parameter and demonstrated that cell-fate decision in response to TGF-β stimulation depends not only on its concentration but also on the time course of its delivery. Because we did not integrate ligand depletion in our model, response predictions were insensitive to TGF-β concentration except for concentrations as low as 0.1 nM (Figure 6A) and we routinely used concentrations of 10 nM as the TGF-β input. When TGF-β depletion was included in our model, both graded short-term and switch-like long-term responses to TGF-β were conserved as reported by Zi *et al.* [19]. However, they were attenuated, suggesting that the presence of TIF1γ does not affect the signal shape, but only the amplitude of TGF-β signaling (Figure S3).

**Figure 7:**
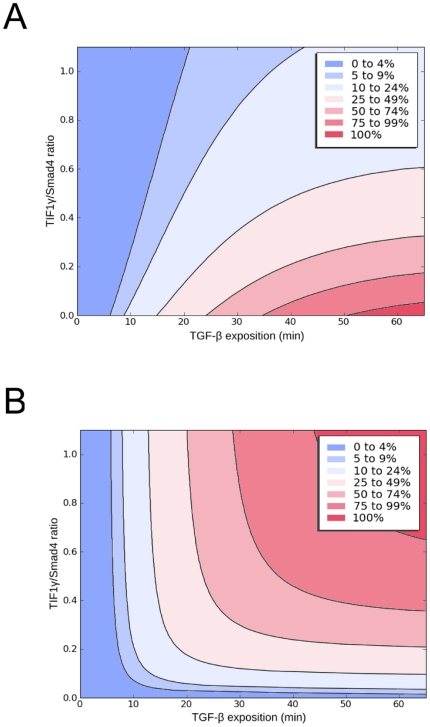
TGF-β time-dependent pS24n and pS2nTIF1γ response profiles as a function of TIF1γ/Smad4 ratios. Results are expressed as percentage of the maximum production of pS24n (A) or pS2nTIF1γ (B).

In contrast, we observed that, in our model, the length of stimulation modified the cell response. This was particularly true for short times (Figure 6B), maximum pS24n complex formation being highly dependent on TIF1γ concentration (Figure 6C and 6D). This indicates that the magnitude of the cellular response to TGF-β depends on both TIF1γ/Smad4 ratios and time-dependent stimulation, predicting a broad range of responses according to TGF-β cellular content and availability in the microenvironment (Figure 7A). Note that the alternative pS2TIF1γ transcription complexes proposed by He *et al.* [7] displayed an opposite profile that required high TIF1γ/Smad4 ratios and longer stimulation times to be fully active (Figure 7B).

In agreement with Zi *et al.* [19], our model showed that periodic short pulses of ligand stimulation yielded an outcome similar to that produced by sustained ligand simulation, whereas an increase in the duration between pulses prevented a continuous response. These observations support the memory concept of ligand-receptor complex (LCR) activity (4A). When TIF1γ was added, the shape of the response was similar, albeit attenuated, suggesting that, in our model, TIF1γ does not affect LCR recycling (Figure S4B).

### Conclusions

Taking into account the seemingly contradictory observations of Smad4-TIF1γ and Smad2/3-TIF1γ interactions, we propose an integrative model based on the formation of Smad2-Smad4-TIF1γ ternary complexes. Validation of our hypotheses by *a posteriori* biological experiments provides strong support for our model, which shows that the TIF1γ/Smad4 ratio serves as a regulator of TGF-β signaling that may affect determination of cell fate. We demonstrate that the response to TGF-β signaling is highly sensitive to TIF1γ/Smad4 ratios, especially for short stimulation times that mediate higher threshold responses. A critical role for the TIF1γ/Smad4 ratio in the regulation of TGF-β signaling is supported by the antagonistic role of TIF1γ and Smad4 in the epithelio-mesenchymal cell transition [12], embryonic patterning and trophoblast stem-cell differentiation [6], suggesting that TIF1γ acts as a negative regulator of higher TGF-β threshold responses.

Our results emphasize the significance of TIF1γ in orchestrating the pleiotropic effects of TGF-β signaling according to the cellular context. Its sensitivity to Smad4 levels and stimulation times suggests that TIF1γ helps define a broad landscape of TGF-β responses. We note that Agricola *et al.* recently proposed a new model for TIF1γ ubiquitin ligase activity that requires binding to histones [22], thus implicating chromatin dynamics in the control of Smad localization at the promoter of TGF-β target genes. According to these results, epigenetic events contribute to the transcriptional regulation of TGF-β target genes *via* acetylation and methylation processes [26]–[28]. In order to understand the complexity of TGF-β-dependent gene regulation and to predict cellular responses, we believe that future models will need to integrate not only the Smad canonical pathway but also Smad-independent pathways and epigenetic events. Because of the lack of quantitative data, such an ambitious goal will require the development of different modeling-based approaches that utilize discrete models [29], [30].

## Supporting Information

Figure S1 **Effects of Smad4 and TIF1γ knockdown on gene expression.** HMEC cells were transfected with Smad4 (siSmad4) or TIF1γ (siTIF1γ) siRNAs and cultured in the presence (+) or absence (−) of TGF-β for the indicated times (days). Controls were cells transfected with non-targeted siRNA (Scr). Smad4 and TIF1γ gene expression was quantified by RT-qPCR. All values were normalized to the amount of HPRT mRNA and expressed in arbitrary units (AU). Results are expressed as the mean+SD of 3 independent experiments.
(PDF)

Figure S2 **Expression of the CDH2 and CDH11 is induced by TGF-β through TIF1γ- and Smad4-dependent pathways.** HMEC cells were transfected with Smad4 (siSmad4) or TIF1γ siRNAs (si TIF1γ) and cultured in the presence (+) or absence (−) of TGF-β for 2 days. Control cells transfected with non-targeted siRNA (Scr). CH2 and CDH11 gene expression was quantified by RT-qPCR. Results are normalized to the amount of mRNA in untreated cells and expressed as the mean+SD of 3 independent experiments.
(PDF)

Figure S3 **TIF1γ does not affect short-term and switch-like long-term responses to TGF-β.** TGF-β depletion was added to the integrated model and modeling analysis of the pS24n response was performed using either increasing concentrations of TGF-β (A) or increasing concentrations of TIF1γ in the presence of 1 nM (B) 5 nM (C) and 10 nM TGF-β (D).
(PDF)

Figure S4 **TIF1γ does not modify the pS24n response to a pulsed exposure to TGF-β.** Model prediction of the pS24n response in the absence (A) or presence (B) of 10 nM TIF1γ to sustained TGF-β (10 nM) stimulation (blue curve), continuous short pulses at 30-minute intervals (green curve) or 3-hour intervals (red curve), as previously described experimentally (Zi et al 2011). We use 10 nM TIF1γ as an average dose of tested concentrations. Concentrations up to 50 nM TIF1γ did not modify the behavior of the signal but only reduced the signal range.
(PDF)

Table S1 **System parameters.**
(PDF)

Table S2 **System of ordinary differential equations.** Equations in black are from Vilar *et al.*, 2006 and Schmierer *et al.*, 2008; equations in red are estimated from biological experiments from Dupont *et al*., 2005, 09 and He *et al*., 2006.
(PDF)

Model S1 **Description of the model in Systems Biology Markup Language (SBML).**
(PDF)
