# Supplementary figures and images for: Dynamic Regulation of Tgf-B Signaling by Tif1γ: A Computational Approach
Source: PLoS One. 2012 Mar 23;7(3):e33761. doi: 10.1371/journal.pone.0033761 (PMC3314286; doi:10.1371/journal.pone.0033761)

**Figure S1**

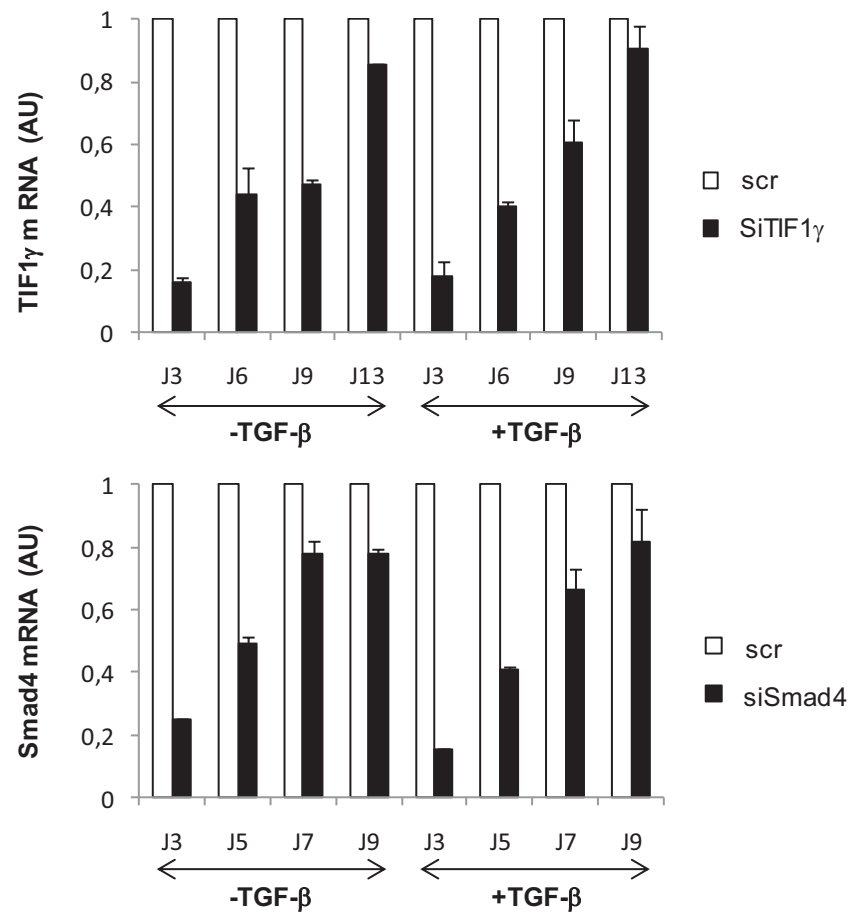

Supplement: Figure S1 — Effects of Smad4 and TIF1γ knockdown on gene expression. HMEC cells were transfected with Smad4 (siSmad4) or TIF1γ (siTIF1γ) siRNAs and cultured in the presence (+) or absence (−) of TGF-β for the indicated times (days). Controls were cells transfected with non-targeted siRNA (Scr). Smad4 and TIF1γ gene expression was quantified by RT-qPCR. All values were normalized to the amount of HPRT mRNA and expressed in arbitrary units (AU). Results are expressed as the mean+SD of 3 independent experiments. (PDF) [file pone.0033761.s001.pdf]

**Figure S2**

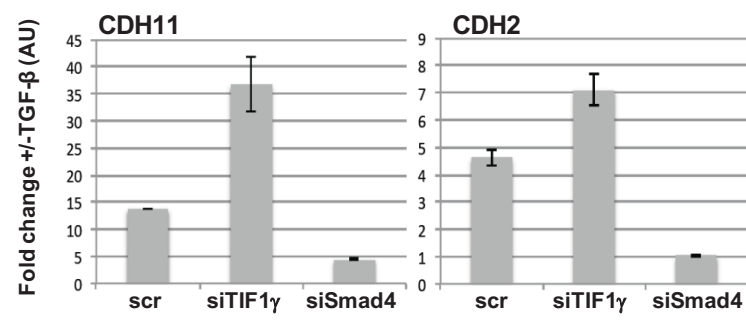

Supplement: Figure S2 — Expression of the CDH2 and CDH11 is induced by TGF-β through TIF1γ- and Smad4-dependent pathways. HMEC cells were transfected with Smad4 (siSmad4) or TIF1γ siRNAs (si TIF1γ) and cultured in the presence (+) or absence (−) of TGF-β for 2 days. Control cells transfected with non-targeted siRNA (Scr). CH2 and CDH11 gene expression was quantified by RT-qPCR. Results are normalized to the amount of mRNA in untreated cells and expressed as the mean+SD of 3 independent experiments. (PDF) [file pone.0033761.s002.pdf]

Figure S3

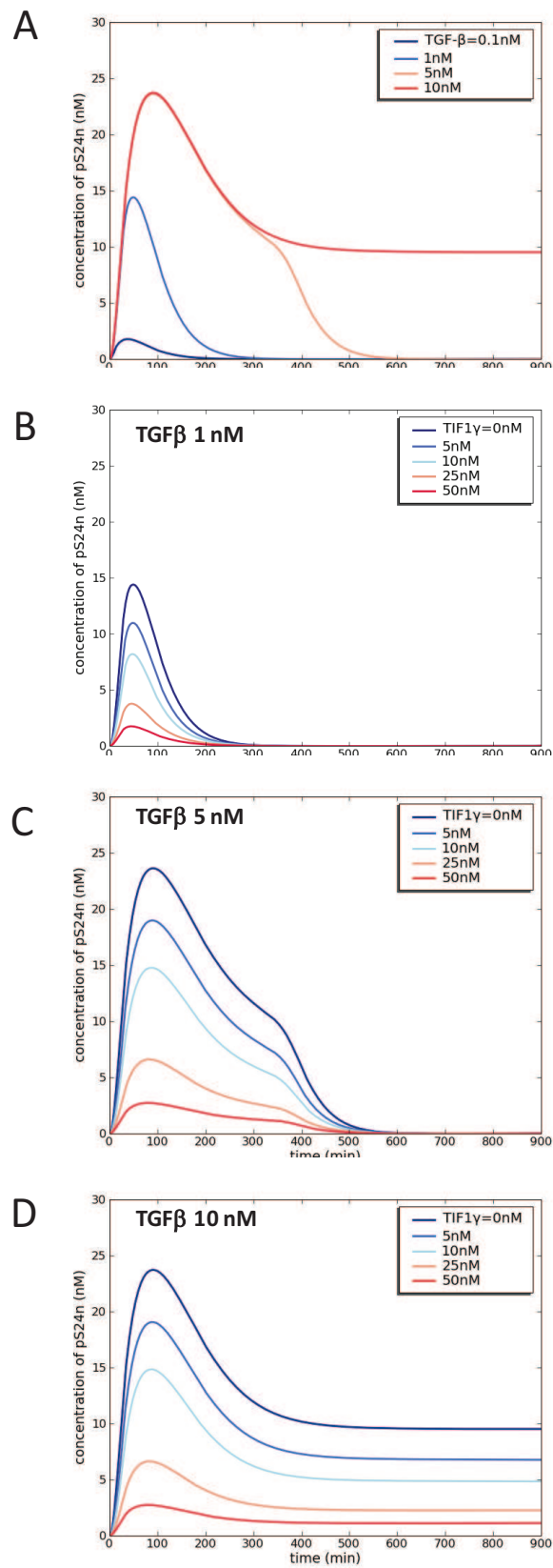

Supplement: Figure S3 — TIF1γ does not affect short-term and switch-like long-term responses to TGF-β. TGF-β depletion was added to the integrated model and modeling analysis of the pS24n response was performed using either increasing concentrations of TGF-β (A) or increasing concentrations of TIF1γ in the presence of 1 nM (B) 5 nM (C) and 10 nM TGF-β (D). (PDF) [file pone.0033761.s003.pdf]

Figure S4

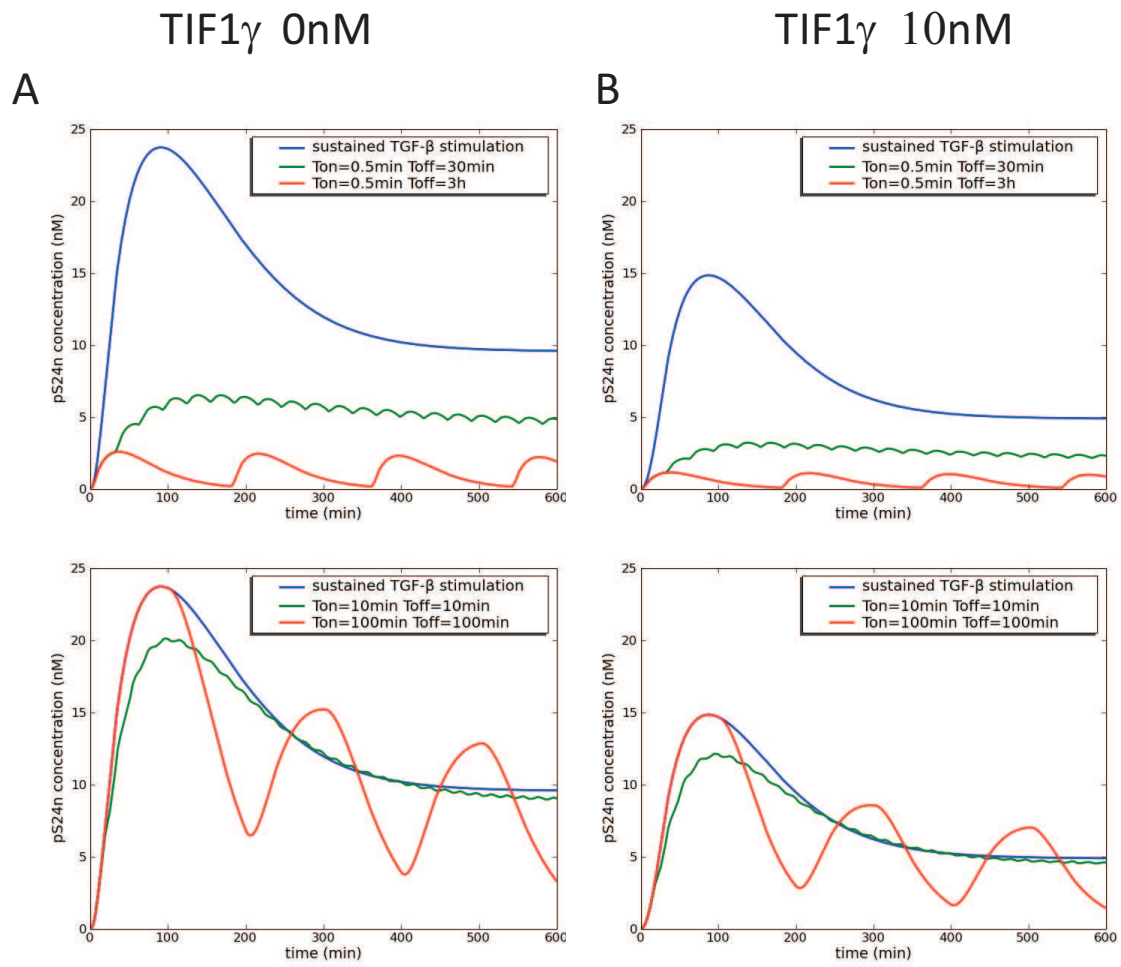

Supplement: Figure S4 — TIF1γ does not modify the pS24n response to a pulsed exposure to TGF-β. Model prediction of the pS24n response in the absence (A) or presence (B) of 10 nM TIF1γ to sustained TGF-β (10 nM) stimulation (blue curve), continuous short pulses at 30-minute intervals (green curve) or 3-hour intervals (red curve), as previously described experimentally (Zi et al 2011). We use 10 nM TIF1γ as an average dose of tested concentrations. Concentrations up to 50 nM TIF1γ did not modify the behavior of the signal but only reduced the signal range. (PDF) [file pone.0033761.s004.pdf]
