# Supplementary material for: Dynamic Regulation of Tgf-B Signaling by Tif1γ: A Computational Approach
Source: PLoS One. 2012 Mar 23;7(3):e33761. doi: 10.1371/journal.pone.0033761 (PMC3314286; doi:10.1371/journal.pone.0033761)
Supplement: Table S1 — System parameters. (PDF) [file pone.0033761.s005.pdf]

Table S1

Symbol	Definition	Value	Reference
S2c	cytoplasmic SMAD2	121.2nM	Schmierer et al, PNAS, 2008
S2n	nuclear SMAD2	57nM	Schmierer et al, PNAS, 2008
S4c	cytoplasmic SMAD4	50.8nM	Schmierer et al, PNAS, 2008
S4n	nuclear SMAD4	50.8nM	Schmierer et al, PNAS, 2008
pS2c	cytoplasmic phospho SMAD2	0nM	Schmierer et al, PNAS, 2008
pS2n	nuclear phospho SMAD2	0nM	Schmierer et al, PNAS, 2008
pS24c	cytoplasmic SMAD2 SMAD4 complex	0nM	Schmierer et al, PNAS, 2008
pS24n	nuclear SMAD2 SMAD4 complex	0nM	Schmierer et al, PNAS, 2008
pS22c	cytoplasmic SMAD2 SMAD2 complex	0nM	Schmierer et al, PNAS, 2008
pS22n	nuclear SMAD2 SMAD2 complex	0nM	Schmierer et al, PNAS, 2008
TGFβ	transforming growth factor beta	0 or 10nM	Schmierer et al, PNAS, 2008
PPase	Phosphatase	1nM	Schmierer et al, PNAS, 2008
RI	TGF type I receptor	3.66nM	Vilar et al, Plos Computational Biology, 2006
RII	TGF type II receptor	3.66nM	Vilar et al, Plos Computational Biology, 2006
LR	ligand receptor I receptor II complex	0nM	Vilar et al, Plos Computational Biology, 2006
RIe	endosomal TGF type I receptor	0nM	Vilar et al, Plos Computational Biology, 2006
RIIe	endosomal TGF type II receptor	0nM	Vilar et al, Plos Computational Biology, 2006
LRe	endosomal ligand receptor I receptor II complex	0nM	Vilar et al, Plos Computational Biology, 2006
TIF1γ	Transcriptional Intermediary Factor 1γ	from 0 to 50nM	Dupont et al, Cell, 2009
FAM	deubiquitinase	10nM	Dupont et al, Cell, 2009
pS24nTIF1γ	nuclear SMAD2 SMAD4 TIF1γ complex	0nM	Dupont et al, Cell, 2009 and He et al, Cell, 2006
pS2nTIF1γ	nuclear phospho SMAD2 TIF1γ complex	0nM	Dupont et al, Cell, 2009 and He et al, Cell, 2006
S4ub c	cytoplasmic ubiquitinate SMAD4	0nM	Dupont et al, Cell, 2009
S4ub n	nuclear ubiquitinate SMAD4	0nM	Dupont et al, Cell, 2009
k_{in}	import rate	$2.6 * 10^{-3} s^{-1}$	Schmierer et al, PNAS, 2008
k_{ex}	export rate	$5.6 * 10^{-2} s^{-1}$	Schmierer et al, PNAS, 2008
k_{phos}	phosphorylation rate	$4.04 * 10^{-4} nM^{-1}.s^{-1}$	Schmierer et al, PNAS, 2008
k_{dephos}	dephosphorylation rate	$7 * 10^{-3} nM^{-1}.s^{-1}$	Schmierer et al, PNAS, 2008
CIF	complex import factor	5.672 no unit	Schmierer et al, PNAS, 2008
k_{on}	Smad complex association rate	$2 * 10^{-3} nM^{-1}.s^{-1}$	Schmierer et al, PNAS, 2008
k_{off}	Smad complex separation rate	$1.6 * 10^{-2} s^{-1}$	Schmierer et al, PNAS, 2008
k_a	Ligand receptor association rate	$1 nM^{-2}.s^{-1}$	Vilar et al, Plos Computational Biology, 2006
k_{cd}	constitutive degradation rate	$4.68 * 10^{-4} s^{-1}$	Vilar et al, Plos Computational Biology, 2006
k_{lid}	ligand induces degradation rate	$4.16 * 10^{-3} s^{-1}$	Vilar et al, Plos Computational Biology, 2006
k_i	internalization rate	$5.55 * 10^{-3} s^{-1}$	Vilar et al, Plos Computational Biology, 2006
k_r	recycling rate	$5.55 * 10^{-4} s^{-1}$	Vilar et al, Plos Computational Biology, 2006
pRI	receptors I production rate	$9.75 * 10^{-5} nM.s^{-1}$	Vilar et al, Plos Computational Biology, 2006
pRII	receptors II production rate	$4.87 * 10^{-5} nM.s^{-1}$	Vilar et al, Plos Computational Biology, 2006
alpha	efficiency of recycling of active receptors	1 no unit	Vilar et al, Plos Computational Biology, 2006
$k_{on\,pS24nTIF1\gamma}$	pS24nTIF1γ complex association rate	$2 * 10^{-3} nM^{-1}.s^{-1}$	Dupont et al, Cell, 2009
$k_{off\,pS24nTIF1\gamma}$	pS24nTIF1γ complex dissociation rate	$1.6 * 10^{-2} s^{-1}$	Dupont et al, Cell, 2009
$k_{off\,pS2nTIF1\gamma}$	pS2nTIF1γ complex dissociation rate	$1.6 * 10^{-2} s^{-1}$	He et al, Cell, 2006
k_{inS4ub}	S4ub import rate	$5.2 * 10^{-3} s^{-1}$	Dupont et al, Cell, 2009
K_{dub}	S4ub c deubiquitination rate	$7 * 10^{-3} nM^{-1}.s^{-1}$	Dupont et al, Cell, 2009
