# Supplementary material for: Dynamic Regulation of Tgf-B Signaling by Tif1γ: A Computational Approach
Source: PLoS One. 2012 Mar 23;7(3):e33761. doi: 10.1371/journal.pone.0033761 (PMC3314286; doi:10.1371/journal.pone.0033761)
Supplement: Table S2 — System of ordinary differential equations. Equations in black are from Vilar et al., 2006 and Schmierer et al., 2008; equations in red are estimated from biological experiments from Dupont et al., 2005, 09 and He et al., 2006. (PDF) [file pone.0033761.s006.pdf]

Table S2

$$
\begin{cases}
\frac{d}{dt}[S4c] = k_{in}[S4n] - k_{in}[S4c] - k_{on}[S4n][pS2n] + k_{off}[pS24c] + k_{dub}[S4ubc][FAM] \\
\frac{d}{dt}[S4n] = k_{in}[S4c] - k_{in}[S4n] - k_{on}[S4n][pS2n] + k_{off}[pS24n] \\
\frac{d}{dt}[S2c] = k_{ex}[S2n] - k_{in}[S2c] - k_{phos}[S2c][LRe] \\
\frac{d}{dt}[S2n] = k_{in}[S2c] - k_{ex}[S2n] + k_{dephos}[S24n][PPase] \\
\frac{d}{dt}[pS2c] = k_{ex}[pS2n] - k_{in}[pS2c] + k_{phos}[S2c][LRe] - k_{on}[pS2c]([S4c] + 2[pS2c]) + k_{off}([pS24c] + 2[pS22c]) \\
\frac{d}{dt}[pS2n] = k_{in}[pS2c] - k_{ex}[pS2n] - k_{dephos}[pS2n][PPase] - k_{on}[pS2n]([S4n] + 2[pS2n]) + k_{off}([pS24n] + 2[pS22n]) \\
\quad + k_{off\,pS2nTIF1\gamma}[pS2nTIF1\gamma] \\
\frac{d}{dt}[pS24c] = k_{on}[pS2c][S4c] - k_{off}[pS24c] - k_{in} * CIF * [pS24c] \\
\frac{d}{dt}[pS24n] = k_{on}[pS2n][S4n] - k_{off}[pS24n] + k_{in} * CIF * [pS24c] - k_{on\,pS24nTIF1\gamma}[pS24n][TIF1\gamma] \\
\frac{d}{dt}[pS22c] = k_{on} * 2[pS2c] - k_{off}[pS22c] - k_{in} * CIF * [pS22c] \\
\frac{d}{dt}[pS22n] = k_{on} * 2[pS2n] - k_{off}[pS22n] + k_{in} * CIF * [pS22c] \\
\frac{d}{dt}[TGF\beta] = 0 \\
\frac{d}{dt}[PPase] = 0 \\
\frac{d}{dt}[LR] = k_a[TGF\beta][RI][RII] - (k_{cd} + k_{lid} + k_i)[LR] \\
\frac{d}{dt}[RI] = pRI - k_a[TGF\beta][RI][RII] - (k_{cd} + k_i)[RI] + k_r[RIe] + alpha * k_r[LRe] \\
\frac{d}{dt}[RII] = pRII - k_a[TGF\beta][RI][RII] - (k_{cd} + k_i)[RII] + k_r[RIIe] + alpha * k_r[LRe] \\
\frac{d}{dt}[RIe] = k_i[RI] - k_r[RIe] \\
\frac{d}{dt}[RIIe] = k_i[RII] - k_r[RIIe] \\
\frac{d}{dt}[LRe] = k_i[LR] - k_r[LRe] \\
\frac{d}{dt}[TIF1\gamma] = -k_{on\,pS24nTIF1\gamma}[pS24n][TIF1\gamma] + k_{off\,pS2nTIF1\gamma}[pS2nTIF1\gamma] \\
\frac{d}{dt}[FAM] = 0 \\
\frac{d}{dt}[pS24nTIF1\gamma] = k_{on\,pS24nTIF1\gamma}[pS24n][TIF1\gamma] - k_{off\,pS24nTIF1\gamma}[pS24nTIF1\gamma] \\
\frac{d}{dt}[pS2nTIF1\gamma] = k_{off\,pS24nTIF1\gamma}[pS24nTIF1\gamma] - k_{off\,pS2nTIF1\gamma}[pS2nTIF1\gamma] \\
\frac{d}{dt}[S4ubc] = k_{in\,S4ub}[S4ubn] - k_{dub}[S4ubc][FAM] \\
\frac{d}{dt}[S4ubn] = k_{off\,pS24nTIF1\gamma}[pS24nTIF1\gamma] - k_{in\,S4ub}[S4ubn]
\end{cases}
$$
