# Supplementary material for: Dynamic Regulation of Tgf-B Signaling by Tif1γ: A Computational Approach
Source: PLoS One. 2012 Mar 23;7(3):e33761. doi: 10.1371/journal.pone.0033761 (PMC3314286; doi:10.1371/journal.pone.0033761)
Supplement: Model S1 — Description of the model in Systems Biology Markup Language (SBML). (PDF) [file pone.0033761.s007.pdf]

```
<?xml version="1.0" encoding="UTF-8"?>
<sbml xmlns="http://www.sbml.org/sbml/level2" metaid="metaid_0000080" level="2" version="1">
  <model metaid="metaid_0000079" id="TGFbeta_regulation_by_TIF1gamma"
name="TGFbeta_regulation_by_TIF1gamma">
  <listOfUnitDefinitions>
    <unitDefinition metaid="metaid_0000081" id="substance">
      <listOfUnits>
        <unit kind="mole" scale="-9"/>
      </listOfUnits>
    </unitDefinition>
    <unitDefinition metaid="metaid_0000082" id="nM" name="nM">
      <listOfUnits>
        <unit kind="mole" scale="-9"/>
        <unit kind="litre" exponent="-1"/>
      </listOfUnits>
    </unitDefinition>
    <unitDefinition metaid="metaid_0000083" id="ps" name="persecond">
      <listOfUnits>
        <unit kind="second" exponent="-1"/>
      </listOfUnits>
    </unitDefinition>
    <unitDefinition metaid="metaid_0000084" id="pnMps" name="pernMpersecond">
      <listOfUnits>
        <unit kind="mole" exponent="-1" scale="-9"/>
        <unit kind="litre"/>
        <unit kind="second" exponent="-1"/>
      </listOfUnits>
    </unitDefinition>
    <unitDefinition metaid="metaid_0000088" id="pnM2ps" name="pernM2persecond">
      <listOfUnits>
        <unit kind="mole" exponent="-2" scale="-9"/>
        <unit kind="litre"/>
        <unit kind="second" exponent="-1"/>
      </listOfUnits>
    </unitDefinition>
    <unitDefinition metaid="metaid_0000085" id="nMps" name="nMpersecond">
      <listOfUnits>
        <unit kind="mole" scale="-9"/>
        <unit kind="litre" exponent="-1"/>
        <unit kind="second" exponent="-1"/>
      </listOfUnits>
    </unitDefinition>
  </listOfUnitDefinitions>
  <listOfCompartments>
    <compartment metaid="metaid_0000047" id="PM" name="Plasma membrane" size="1">
      <annotation>
        <rdf:RDF xmlns:rdf="http://www.w3.org/1999/02/22-rdf-syntax-ns#" xmlns:dc="http://purl.org/dc/
elements/1.1/" xmlns:dcterms="http://purl.org/dc/terms/" xmlns:vCard="http://www.w3.org/2001/vcard-
rdf/3.0#" xmlns:bqbiol="http://biomodels.net/biology-qualifiers/" xmlns:bqmodel="http://biomodels.net/
model-qualifiers/">
          <rdf:Description rdf:about="#metaid_0000047">
            <bqbiol:is>
              <rdf:Bag>
                <rdf:li rdf:resource="urn:miriam:obo.go:GO%3A0005886"/>
              </rdf:Bag>
            </bqbiol:is>
          </rdf:Description>
        </rdf:RDF>
      </annotation>
    </compartment>
    <compartment metaid="metaid_0000048" id="Endosome" name="Endosome" size="1" outside="PM">
      <annotation>
        <rdf:RDF xmlns:rdf="http://www.w3.org/1999/02/22-rdf-syntax-ns#" xmlns:dc="http://purl.org/dc/
elements/1.1/" xmlns:dcterms="http://purl.org/dc/terms/" xmlns:vCard="http://www.w3.org/2001/vcard-
rdf/3.0#" xmlns:bqbiol="http://biomodels.net/biology-qualifiers/" xmlns:bqmodel="http://biomodels.net/
model-qualifiers/">
          <rdf:Description rdf:about="#metaid_0000048">
            <bqbiol:is>
              <rdf:Bag>
                <rdf:li rdf:resource="urn:miriam:obo.go:GO%3A0005768"/>
              </rdf:Bag>
            </bqbiol:is>
```

```xml
          </rdf:Description>
        </rdf:RDF>
      </annotation>
    </compartment>
    <compartment metaid="metaid_0000049" id="nucleus" name="Nuc" size="1e-12">
      <annotation>
        <rdf:RDF xmlns:rdf="http://www.w3.org/1999/02/22-rdf-syntax-ns#" xmlns:dc="http://purl.org/dc/
elements/1.1/" xmlns:dcterms="http://purl.org/dc/terms/" xmlns:vCard="http://www.w3.org/2001/vcard-
rdf/3.0#" xmlns:bqbiol="http://biomodels.net/biology-qualifiers/" xmlns:bqmodel="http://biomodels.net/
model-qualifiers/">
          <rdf:Description rdf:about="#metaid_0000049">
            <bqbiol:is>
              <rdf:Bag>
                <rdf:li rdf:resource="urn:miriam:obo.go:GO%3A0005634"/>
              </rdf:Bag>
            </bqbiol:is>
          </rdf:Description>
        </rdf:RDF>
      </annotation>
    </compartment>
    <compartment metaid="metaid_0000050" id="cytosol" name="Cyt" size="2.27e-12">
      <annotation>
        <rdf:RDF xmlns:rdf="http://www.w3.org/1999/02/22-rdf-syntax-ns#" xmlns:dc="http://purl.org/dc/
elements/1.1/" xmlns:dcterms="http://purl.org/dc/terms/" xmlns:vCard="http://www.w3.org/2001/vcard-
rdf/3.0#" xmlns:bqbiol="http://biomodels.net/biology-qualifiers/" xmlns:bqmodel="http://biomodels.net/
model-qualifiers/">
          <rdf:Description rdf:about="#metaid_0000050">
            <bqmodel:is>
              <rdf:Bag>
                <rdf:li rdf:resource="urn:miriam:obo.go:GO%3A0005737"/>
              </rdf:Bag>
            </bqmodel:is>
          </rdf:Description>
        </rdf:RDF>
      </annotation>
    </compartment>
  </listOfCompartments>
  <listOfSpecies>
    <species metaid="metaid_0000001" id="PPase" name="PPase" compartment="nucleus"
initialConcentration="1" boundaryCondition="true" constant="true">
      <annotation>
        <rdf:RDF xmlns:rdf="http://www.w3.org/1999/02/22-rdf-syntax-ns#" xmlns:dc="http://purl.org/dc/
elements/1.1/" xmlns:dcterms="http://purl.org/dc/terms/" xmlns:vCard="http://www.w3.org/2001/vcard-
rdf/3.0#" xmlns:bqbiol="http://biomodels.net/biology-qualifiers/" xmlns:bqmodel="http://biomodels.net/
model-qualifiers/">
          <rdf:Description rdf:about="#metaid_0000001">
            <bqbiol:isVersionOf>
              <rdf:Bag>
                <rdf:li rdf:resource="urn:miriam:uniprot:P35813"/>
                <rdf:li rdf:resource="urn:miriam:obo.go:GO%3A0004721"/>
              </rdf:Bag>
            </bqbiol:isVersionOf>
          </rdf:Description>
        </rdf:RDF>
      </annotation>
    </species>
    <species metaid="metaid_0000002" id="S2n" name="Smad2_n" compartment="nucleus"
initialConcentration="57" hasOnlySubstanceUnits="false">
      <annotation>
        <rdf:RDF xmlns:rdf="http://www.w3.org/1999/02/22-rdf-syntax-ns#" xmlns:dc="http://purl.org/dc/
elements/1.1/" xmlns:dcterms="http://purl.org/dc/terms/" xmlns:vCard="http://www.w3.org/2001/vcard-
rdf/3.0#" xmlns:bqbiol="http://biomodels.net/biology-qualifiers/" xmlns:bqmodel="http://biomodels.net/
model-qualifiers/">
          <rdf:Description rdf:about="#metaid_0000002">
            <bqbiol:isVersionOf>
              <rdf:Bag>
                <rdf:li rdf:resource="urn:miriam:uniprot:Q15796"/>
              </rdf:Bag>
            </bqbiol:isVersionOf>
          </rdf:Description>
        </rdf:RDF>
      </annotation>
```

```
    </species>
    <species metaid="metaid_0000003" id="pS2n" name="pSmad2_n" compartment="nucleus"
initialConcentration="0" hasOnlySubstanceUnits="false">
      <annotation>
        <rdf:RDF xmlns:rdf="http://www.w3.org/1999/02/22-rdf-syntax-ns#" xmlns:dc="http://purl.org/dc/
elements/1.1/" xmlns:dcterms="http://purl.org/dc/terms/" xmlns:vCard="http://www.w3.org/2001/vcard-
rdf/3.0#" xmlns:bqbiol="http://biomodels.net/biology-qualifiers/" xmlns:bqmodel="http://biomodels.net/
model-qualifiers/">
          <rdf:Description rdf:about="#metaid_0000003">
            <bqbiol:isVersionOf>
              <rdf:Bag>
                <rdf:li rdf:resource="urn:miriam:uniprot:Q15796"/>
                <rdf:li rdf:resource="urn:miriam:kegg.compound:C00562"/>
              </rdf:Bag>
            </bqbiol:isVersionOf>
          </rdf:Description>
        </rdf:RDF>
      </annotation>
    </species>
  <species metaid="metaid_0000004" id="pS22n" name="pSmad2/pSmad2_n" compartment="nucleus"
initialConcentration="0" hasOnlySubstanceUnits="false">
      <annotation>
        <rdf:RDF xmlns:rdf="http://www.w3.org/1999/02/22-rdf-syntax-ns#" xmlns:dc="http://purl.org/dc/
elements/1.1/" xmlns:dcterms="http://purl.org/dc/terms/" xmlns:vCard="http://www.w3.org/2001/vcard-
rdf/3.0#" xmlns:bqbiol="http://biomodels.net/biology-qualifiers/" xmlns:bqmodel="http://biomodels.net/
model-qualifiers/">
          <rdf:Description rdf:about="#metaid_0000004">
            <bqbiol:isVersionOf>
              <rdf:Bag>
                <rdf:li rdf:resource="urn:miriam:obo.go:GO%3A0043234"/>
              </rdf:Bag>
            </bqbiol:isVersionOf>
            <bqbiol:hasPart>
              <rdf:Bag>
                <rdf:li rdf:resource="urn:miriam:uniprot:Q15796"/>
              </rdf:Bag>
            </bqbiol:hasPart>
            <bqbiol:hasVersion>
              <rdf:Bag>
                <rdf:li rdf:resource="urn:miriam:kegg.compound:C00562"/>
              </rdf:Bag>
            </bqbiol:hasVersion>
          </rdf:Description>
        </rdf:RDF>
      </annotation>
    </species>
    <species metaid="metaid_0000005" id="pS24n" name="pSmad2/Smad4_n" compartment="nucleus"
initialConcentration="0" hasOnlySubstanceUnits="false">
      <annotation>
        <rdf:RDF xmlns:rdf="http://www.w3.org/1999/02/22-rdf-syntax-ns#" xmlns:dc="http://purl.org/dc/
elements/1.1/" xmlns:dcterms="http://purl.org/dc/terms/" xmlns:vCard="http://www.w3.org/2001/vcard-
rdf/3.0#" xmlns:bqbiol="http://biomodels.net/biology-qualifiers/" xmlns:bqmodel="http://biomodels.net/
model-qualifiers/">
          <rdf:Description rdf:about="#metaid_0000005">
            <bqbiol:isVersionOf>
              <rdf:Bag>
                <rdf:li rdf:resource="urn:miriam:obo.go:GO%3A0043234"/>
              </rdf:Bag>
            </bqbiol:isVersionOf>
            <bqbiol:hasPart>
              <rdf:Bag>
                <rdf:li rdf:resource="urn:miriam:uniprot:Q15796"/>
                <rdf:li rdf:resource="urn:miriam:uniprot:Q13485"/>
              </rdf:Bag>
            </bqbiol:hasPart>
            <bqbiol:hasVersion>
              <rdf:Bag>
                <rdf:li rdf:resource="urn:miriam:kegg.compound:C00562"/>
              </rdf:Bag>
            </bqbiol:hasVersion>
          </rdf:Description>
        </rdf:RDF>
```

```xml
      </annotation>
    </species>
    <species metaid="metaid_0000006" id="S4n" name="Smad4_n" compartment="nucleus"
initialConcentration="50.8" hasOnlySubstanceUnits="false">
      <annotation>
        <rdf:RDF xmlns:rdf="http://www.w3.org/1999/02/22-rdf-syntax-ns#" xmlns:dc="http://purl.org/dc/
elements/1.1/" xmlns:dcterms="http://purl.org/dc/terms/" xmlns:vCard="http://www.w3.org/2001/vcard-
rdf/3.0#" xmlns:bqbiol="http://biomodels.net/biology-qualifiers/" xmlns:bqmodel="http://biomodels.net/
model-qualifiers/">
          <rdf:Description rdf:about="#metaid_0000006">
            <bqbiol:isVersionOf>
              <rdf:Bag>
                <rdf:li rdf:resource="urn:miriam:uniprot:Q13485"/>
              </rdf:Bag>
            </bqbiol:isVersionOf>
          </rdf:Description>
        </rdf:RDF>
      </annotation>
    </species>
   <species metaid="metaid_0000007" id="pS22c" name="pSmad2/pSmad2_c" compartment="cytosol"
initialConcentration="0" hasOnlySubstanceUnits="false">
      <annotation>
        <rdf:RDF xmlns:rdf="http://www.w3.org/1999/02/22-rdf-syntax-ns#" xmlns:dc="http://purl.org/dc/
elements/1.1/" xmlns:dcterms="http://purl.org/dc/terms/" xmlns:vCard="http://www.w3.org/2001/vcard-
rdf/3.0#" xmlns:bqbiol="http://biomodels.net/biology-qualifiers/" xmlns:bqmodel="http://biomodels.net/
model-qualifiers/">
          <rdf:Description rdf:about="#metaid_0000007">
            <bqbiol:isVersionOf>
              <rdf:Bag>
                <rdf:li rdf:resource="urn:miriam:obo.go:GO%3A0043234"/>
              </rdf:Bag>
            </bqbiol:isVersionOf>
            <bqbiol:hasPart>
              <rdf:Bag>
                <rdf:li rdf:resource="urn:miriam:uniprot:Q15796"/>
              </rdf:Bag>
            </bqbiol:hasPart>
            <bqbiol:hasVersion>
              <rdf:Bag>
                <rdf:li rdf:resource="urn:miriam:kegg.compound:C00562"/>
              </rdf:Bag>
            </bqbiol:hasVersion>
          </rdf:Description>
        </rdf:RDF>
      </annotation>
    </species>
    <species metaid="metaid_0000008" id="pS24c" name="pSmad2/Smad4_c" compartment="cytosol"
initialConcentration="0" hasOnlySubstanceUnits="false">
      <annotation>
        <rdf:RDF xmlns:rdf="http://www.w3.org/1999/02/22-rdf-syntax-ns#" xmlns:dc="http://purl.org/dc/
elements/1.1/" xmlns:dcterms="http://purl.org/dc/terms/" xmlns:vCard="http://www.w3.org/2001/vcard-
rdf/3.0#" xmlns:bqbiol="http://biomodels.net/biology-qualifiers/" xmlns:bqmodel="http://biomodels.net/
model-qualifiers/">
          <rdf:Description rdf:about="#metaid_0000008">
            <bqbiol:isVersionOf>
              <rdf:Bag>
                <rdf:li rdf:resource="urn:miriam:obo.go:GO%3A0043234"/>
              </rdf:Bag>
            </bqbiol:isVersionOf>
            <bqbiol:hasPart>
              <rdf:Bag>
                <rdf:li rdf:resource="urn:miriam:uniprot:Q15796"/>
                <rdf:li rdf:resource="urn:miriam:uniprot:Q13485"/>
              </rdf:Bag>
            </bqbiol:hasPart>
            <bqbiol:hasVersion>
              <rdf:Bag>
                <rdf:li rdf:resource="urn:miriam:kegg.compound:C00562"/>
              </rdf:Bag>
            </bqbiol:hasVersion>
          </rdf:Description>
        </rdf:RDF>
```

```xml
      </annotation>
    </species>
    <species metaid="metaid_0000009" id="S4c" name="Smad4_c" compartment="cytosol"
initialConcentration="50.8" hasOnlySubstanceUnits="false">
      <annotation>
        <rdf:RDF xmlns:rdf="http://www.w3.org/1999/02/22-rdf-syntax-ns#" xmlns:dc="http://purl.org/dc/
elements/1.1/" xmlns:dcterms="http://purl.org/dc/terms/" xmlns:vCard="http://www.w3.org/2001/vcard-
rdf/3.0#" xmlns:bqbiol="http://biomodels.net/biology-qualifiers/" xmlns:bqmodel="http://biomodels.net/
model-qualifiers/">
          <rdf:Description rdf:about="#metaid_0000009">
            <bqbiol:isVersionOf>
              <rdf:Bag>
                <rdf:li rdf:resource="urn:miriam:uniprot:Q13485"/>
              </rdf:Bag>
            </bqbiol:isVersionOf>
          </rdf:Description>
        </rdf:RDF>
      </annotation>
    </species>
    <species metaid="metaid_0000010" id="S2c" name="Smad2_c" compartment="cytosol"
initialConcentration="121.2" hasOnlySubstanceUnits="false">
      <annotation>
        <rdf:RDF xmlns:rdf="http://www.w3.org/1999/02/22-rdf-syntax-ns#" xmlns:dc="http://purl.org/dc/
elements/1.1/" xmlns:dcterms="http://purl.org/dc/terms/" xmlns:vCard="http://www.w3.org/2001/vcard-
rdf/3.0#" xmlns:bqbiol="http://biomodels.net/biology-qualifiers/" xmlns:bqmodel="http://biomodels.net/
model-qualifiers/">
          <rdf:Description rdf:about="#metaid_0000010">
            <bqbiol:isVersionOf>
              <rdf:Bag>
                <rdf:li rdf:resource="urn:miriam:uniprot:Q15796"/>
              </rdf:Bag>
            </bqbiol:isVersionOf>
          </rdf:Description>
        </rdf:RDF>
      </annotation>
    </species>
    <species metaid="metaid_0000011" id="pS2c" name="pSmad2_c" compartment="cytosol"
initialConcentration="0" hasOnlySubstanceUnits="false">
      <annotation>
        <rdf:RDF xmlns:rdf="http://www.w3.org/1999/02/22-rdf-syntax-ns#" xmlns:dc="http://purl.org/dc/
elements/1.1/" xmlns:dcterms="http://purl.org/dc/terms/" xmlns:vCard="http://www.w3.org/2001/vcard-
rdf/3.0#" xmlns:bqbiol="http://biomodels.net/biology-qualifiers/" xmlns:bqmodel="http://biomodels.net/
model-qualifiers/">
          <rdf:Description rdf:about="#metaid_0000011">
            <bqbiol:isVersionOf>
              <rdf:Bag>
                <rdf:li rdf:resource="urn:miriam:uniprot:Q15796"/>
                <rdf:li rdf:resource="urn:miriam:kegg.compound:C00562"/>
              </rdf:Bag>
            </bqbiol:isVersionOf>
          </rdf:Description>
        </rdf:RDF>
      </annotation>
    </species>
  <species metaid="metaid_0000012" id="TGFb" name="TGFb" compartment="PM" initialConcentration="10"
boundaryCondition="true" constant="true">
      <annotation>
        <rdf:RDF xmlns:rdf="http://www.w3.org/1999/02/22-rdf-syntax-ns#" xmlns:dc="http://purl.org/dc/
elements/1.1/" xmlns:dcterms="http://purl.org/dc/terms/" xmlns:vCard="http://www.w3.org/2001/vcard-
rdf/3.0#" xmlns:bqbiol="http://biomodels.net/biology-qualifiers/" xmlns:bqmodel="http://biomodels.net/
model-qualifiers/">
          <rdf:Description rdf:about="#metaid_0000012">
            <bqbiol:hasVersion>
              <rdf:Bag>
                <rdf:li rdf:resource="urn:miriam:uniprot:P01137"/>
                <rdf:li rdf:resource="urn:miriam:uniprot:P61812"/>
                <rdf:li rdf:resource="urn:miriam:uniprot:P10600"/>
              </rdf:Bag>
            </bqbiol:hasVersion>
          </rdf:Description>
        </rdf:RDF>
      </annotation>
```

```
    </species>
  <species metaid="metaid_0000013" id="RI" name="Receptor 1" compartment="PM" initialAmount="3.66"
hasOnlySubstanceUnits="false">
      <annotation>
        <rdf:RDF xmlns:rdf="http://www.w3.org/1999/02/22-rdf-syntax-ns#" xmlns:dc="http://purl.org/dc/
elements/1.1/" xmlns:dcterms="http://purl.org/dc/terms/" xmlns:vCard="http://www.w3.org/2001/vcard-
rdf/3.0#" xmlns:bqbiol="http://biomodels.net/biology-qualifiers/" xmlns:bqmodel="http://biomodels.net/
model-qualifiers/">
          <rdf:Description rdf:about="#metaid_0000013">
            <bqbiol:isVersionOf>
              <rdf:Bag>
                <rdf:li rdf:resource="urn:miriam:uniprot:P36897"/>
              </rdf:Bag>
            </bqbiol:isVersionOf>
          </rdf:Description>
        </rdf:RDF>
      </annotation>
    </species>
    <species metaid="metaid_0000014" id="RII" name="Receptor 2" compartment="PM" initialAmount="3.66"
hasOnlySubstanceUnits="false">
      <annotation>
        <rdf:RDF xmlns:rdf="http://www.w3.org/1999/02/22-rdf-syntax-ns#" xmlns:dc="http://purl.org/dc/
elements/1.1/" xmlns:dcterms="http://purl.org/dc/terms/" xmlns:vCard="http://www.w3.org/2001/vcard-
rdf/3.0#" xmlns:bqbiol="http://biomodels.net/biology-qualifiers/" xmlns:bqmodel="http://biomodels.net/
model-qualifiers/">
          <rdf:Description rdf:about="#metaid_0000014">
            <bqbiol:isVersionOf>
              <rdf:Bag>
                <rdf:li rdf:resource="urn:miriam:uniprot:P37173"/>
              </rdf:Bag>
            </bqbiol:isVersionOf>
          </rdf:Description>
        </rdf:RDF>
      </annotation>
    </species>
    <species metaid="metaid_0000015" id="LR" name="ligand receptor complex-plasma membrane"
compartment="PM" initialAmount="0" hasOnlySubstanceUnits="false">
      <annotation>
        <rdf:RDF xmlns:rdf="http://www.w3.org/1999/02/22-rdf-syntax-ns#" xmlns:dc="http://purl.org/dc/
elements/1.1/" xmlns:dcterms="http://purl.org/dc/terms/" xmlns:vCard="http://www.w3.org/2001/vcard-
rdf/3.0#" xmlns:bqbiol="http://biomodels.net/biology-qualifiers/" xmlns:bqmodel="http://biomodels.net/
model-qualifiers/">
          <rdf:Description rdf:about="#metaid_0000015">
            <bqbiol:hasPart>
              <rdf:Bag>
                <rdf:li rdf:resource="urn:miriam:uniprot:P37173"/>
                <rdf:li rdf:resource="urn:miriam:uniprot:P36897"/>
                <rdf:li rdf:resource="urn:miriam:uniprot:P01137"/>
              </rdf:Bag>
            </bqbiol:hasPart>
          </rdf:Description>
        </rdf:RDF>
      </annotation>
    </species>
    <species metaid="metaid_0000016" id="LRe" name="ligand receptor complex-endosome"
compartment="Endosome" initialAmount="0" hasOnlySubstanceUnits="false">
      <annotation>
        <rdf:RDF xmlns:rdf="http://www.w3.org/1999/02/22-rdf-syntax-ns#" xmlns:dc="http://purl.org/dc/
elements/1.1/" xmlns:dcterms="http://purl.org/dc/terms/" xmlns:vCard="http://www.w3.org/2001/vcard-
rdf/3.0#" xmlns:bqbiol="http://biomodels.net/biology-qualifiers/" xmlns:bqmodel="http://biomodels.net/
model-qualifiers/">
          <rdf:Description rdf:about="#metaid_0000016">
            <bqbiol:hasPart>
              <rdf:Bag>
                <rdf:li rdf:resource="urn:miriam:uniprot:P37173"/>
                <rdf:li rdf:resource="urn:miriam:uniprot:P36897"/>
                <rdf:li rdf:resource="urn:miriam:uniprot:P01137"/>
              </rdf:Bag>
            </bqbiol:hasPart>
          </rdf:Description>
        </rdf:RDF>
      </annotation>
```

```
    </species>
    <species metaid="metaid_0000017" id="RIe" name="Receptor 1-endosome" compartment="Endosome"
initialAmount="0" hasOnlySubstanceUnits="false">
      <annotation>
        <rdf:RDF xmlns:rdf="http://www.w3.org/1999/02/22-rdf-syntax-ns#" xmlns:dc="http://purl.org/dc/
elements/1.1/" xmlns:dcterms="http://purl.org/dc/terms/" xmlns:vCard="http://www.w3.org/2001/vcard-
rdf/3.0#" xmlns:bqbiol="http://biomodels.net/biology-qualifiers/" xmlns:bqmodel="http://biomodels.net/
model-qualifiers/">
          <rdf:Description rdf:about="#metaid_0000017">
            <bqbiol:isVersionOf>
              <rdf:Bag>
                <rdf:li rdf:resource="urn:miriam:uniprot:P36897"/>
              </rdf:Bag>
            </bqbiol:isVersionOf>
          </rdf:Description>
        </rdf:RDF>
      </annotation>
    </species>
    <species metaid="metaid_0000018" id="RIIe" name="Receptor 2 endosome" compartment="Endosome"
initialAmount="0" hasOnlySubstanceUnits="false">
      <annotation>
        <rdf:RDF xmlns:rdf="http://www.w3.org/1999/02/22-rdf-syntax-ns#" xmlns:dc="http://purl.org/dc/
elements/1.1/" xmlns:dcterms="http://purl.org/dc/terms/" xmlns:vCard="http://www.w3.org/2001/vcard-
rdf/3.0#" xmlns:bqbiol="http://biomodels.net/biology-qualifiers/" xmlns:bqmodel="http://biomodels.net/
model-qualifiers/">
          <rdf:Description rdf:about="#metaid_0000018">
            <bqbiol:isVersionOf>
              <rdf:Bag>
                <rdf:li rdf:resource="urn:miriam:uniprot:P37173"/>
              </rdf:Bag>
            </bqbiol:isVersionOf>
          </rdf:Description>
        </rdf:RDF>
      </annotation>
    </species>
    <species metaid="metaid_0000019" id="TIF1gamma" name="TIF1-gamma" compartment="nucleus"
initialAmount="0" hasOnlySubstanceUnits="false">
      <annotation>
        <rdf:RDF xmlns:rdf="http://www.w3.org/1999/02/22-rdf-syntax-ns#" xmlns:dc="http://purl.org/dc/
elements/1.1/" xmlns:dcterms="http://purl.org/dc/terms/" xmlns:vCard="http://www.w3.org/2001/vcard-
rdf/3.0#" xmlns:bqbiol="http://biomodels.net/biology-qualifiers/" xmlns:bqmodel="http://biomodels.net/
model-qualifiers/">
          <rdf:Description rdf:about="#metaid_0000019">
            <bqbiol:isVersionOf>
              <rdf:Bag>
                <rdf:li rdf:resource="urn:miriam:uniprot:Q9UPN9"/>
              </rdf:Bag>
            </bqbiol:isVersionOf>
          </rdf:Description>
        </rdf:RDF>
      </annotation>
    </species>
    <species metaid="metaid_0000020" id="FAM" name="FAM" compartment="cytosol" initialAmount="0"
hasOnlySubstanceUnits="false" >
      <annotation>
        <rdf:RDF xmlns:rdf="http://www.w3.org/1999/02/22-rdf-syntax-ns#" xmlns:dc="http://purl.org/dc/
elements/1.1/" xmlns:dcterms="http://purl.org/dc/terms/" xmlns:vCard="http://www.w3.org/2001/vcard-
rdf/3.0#" xmlns:bqbiol="http://biomodels.net/biology-qualifiers/" xmlns:bqmodel="http://biomodels.net/
model-qualifiers/">
          <rdf:Description rdf:about="#metaid_0000020">
            <bqbiol:isVersionOf>
              <rdf:Bag>
                <rdf:li rdf:resource="urn:miriam:uniprot:Q93008"/>
              </rdf:Bag>
            </bqbiol:isVersionOf>
          </rdf:Description>
        </rdf:RDF>
      </annotation>
    </species>
    <species metaid="metaid_0000021" id="pS24nTIF1gamma" name="pSmad2/Smad4_n/TIF1-gamma"
compartment="nucleus" initialAmount="0" hasOnlySubstanceUnits="false">
      <annotation>
```

```
        <rdf:RDF xmlns:rdf="http://www.w3.org/1999/02/22-rdf-syntax-ns#" xmlns:dc="http://purl.org/dc/elements/1.1/" xmlns:dcterms="http://purl.org/dc/terms/" xmlns:vCard="http://www.w3.org/2001/vcard-rdf/3.0#" xmlns:bqbiol="http://biomodels.net/biology-qualifiers/" xmlns:bqmodel="http://biomodels.net/model-qualifiers/">
          <rdf:Description rdf:about="#metaid_0000021">
            <bqbiol:hasPart>
              <rdf:Bag>
                <rdf:li rdf:resource="urn:miriam:uniprot:Q15796"/>
                <rdf:li rdf:resource="urn:miriam:uniprot:Q13485"/>
                <rdf:li rdf:resource="urn:miriam:uniprot:Q9UPN9"/>
              </rdf:Bag>
            </bqbiol:hasPart>
          </rdf:Description>
        </rdf:RDF>
      </annotation>
    </species>
    <species metaid="metaid_0000022" id="pS2nTIF1gamma" name="pSmad2_n/TIF1-gamma" compartment="nucleus" initialAmount="0" hasOnlySubstanceUnits="false">
      <annotation>
        <rdf:RDF xmlns:rdf="http://www.w3.org/1999/02/22-rdf-syntax-ns#" xmlns:dc="http://purl.org/dc/elements/1.1/" xmlns:dcterms="http://purl.org/dc/terms/" xmlns:vCard="http://www.w3.org/2001/vcard-rdf/3.0#" xmlns:bqbiol="http://biomodels.net/biology-qualifiers/" xmlns:bqmodel="http://biomodels.net/model-qualifiers/">
          <rdf:Description rdf:about="#metaid_0000022">
            <bqbiol:hasPart>
              <rdf:Bag>
                <rdf:li rdf:resource="urn:miriam:uniprot:Q15796"/>
                <rdf:li rdf:resource="urn:miriam:uniprot:Q9UPN9"/>
              </rdf:Bag>
            </bqbiol:hasPart>
          </rdf:Description>
        </rdf:RDF>
      </annotation>
    </species>
   <species metaid="metaid_0000023" id="S4ub_n" name="nuclear ubiquitinate Smad4" compartment="nucleus" initialConcentration="0" hasOnlySubstanceUnits="false">
      <annotation>
        <rdf:RDF xmlns:rdf="http://www.w3.org/1999/02/22-rdf-syntax-ns#" xmlns:dc="http://purl.org/dc/elements/1.1/" xmlns:dcterms="http://purl.org/dc/terms/" xmlns:vCard="http://www.w3.org/2001/vcard-rdf/3.0#" xmlns:bqbiol="http://biomodels.net/biology-qualifiers/" xmlns:bqmodel="http://biomodels.net/model-qualifiers/">
          <rdf:Description rdf:about="#metaid_0000023">
            <bqbiol:isVersionOf>
              <rdf:Bag>
                <rdf:li rdf:resource="urn:miriam:uniprot:Q13485"/>
                <rdf:li rdf:resource="urn:miriam:kegg.compound:C00496"/>
              </rdf:Bag>
            </bqbiol:isVersionOf>
          </rdf:Description>
        </rdf:RDF>
      </annotation>
    </species>
   <species metaid="metaid_0000024" id="S4ub_c" name="cytoplasmic ubiquitinate Smad4" compartment="cytosol" initialConcentration="0" hasOnlySubstanceUnits="false">
      <annotation>
        <rdf:RDF xmlns:rdf="http://www.w3.org/1999/02/22-rdf-syntax-ns#" xmlns:dc="http://purl.org/dc/elements/1.1/" xmlns:dcterms="http://purl.org/dc/terms/" xmlns:vCard="http://www.w3.org/2001/vcard-rdf/3.0#" xmlns:bqbiol="http://biomodels.net/biology-qualifiers/" xmlns:bqmodel="http://biomodels.net/model-qualifiers/">
          <rdf:Description rdf:about="#metaid_0000024">
            <bqbiol:isVersionOf>
              <rdf:Bag>
                <rdf:li rdf:resource="urn:miriam:uniprot:Q13485"/>
                <rdf:li rdf:resource="urn:miriam:kegg.compound:C00496"/>
              </rdf:Bag>
            </bqbiol:isVersionOf>
          </rdf:Description>
        </rdf:RDF>
      </annotation>
    </species>
  </listOfSpecies>
 <listOfParameters>
```

```xml
    <parameter metaid="metaid_0000025" id="ka" value="1" units="pnM2ps"/>
    <parameter metaid="metaid_0000027" id="kcd" value="4.68e-04" units="ps"/>
    <parameter metaid="metaid_0000028" id="klid" value="4.16e-03" units="ps"/>
    <parameter metaid="metaid_0000029" id="ki" value="5.55e-03" units="ps"/>
    <parameter metaid="metaid_0000030" id="pRI" value="9.75e-05" units="nMps"/>
    <parameter metaid="metaid_0000031" id="kr" value="5.55e-04" units="ps"/>
    <parameter metaid="metaid_0000032" id="alpha" value="1" units="dimensionless"/>
    <parameter metaid="metaid_0000033" id="pRII" value="4.87e-05" units="nMps"/>
    <parameter metaid="metaid_0000034" id="kin" name="kin (import rate for monomeric Smads)"
value="2.6e-03" units="ps"/>
    <parameter metaid="metaid_0000035" id="kex" name="kex (export rate for monomeric Smads)"
value="5.6e-02" units="ps"/>
    <parameter metaid="metaid_0000036" id="kphos" name="kphos (phosphorylation rate)" value="4.04e-04"
units="pnMps"/>
    <parameter metaid="metaid_0000037" id="kdephos" name="kdephos (dephosphorylation rate)"
value="7.10e-03" units="pnMps"/>
    <parameter metaid="metaid_0000038" id="kin_CIF" name="kin*CIF (Complex import rate)"
value="1.48e-02" units="ps"/>
    <parameter metaid="metaid_0000039" id="kon" name="kon (Smad complex on-rate)" value="2e-03"
units="pnMps"/>
    <parameter metaid="metaid_0000040" id="koff" name="koff (Smad complex off-rate)" value="1.6e-02"
units="ps"/>
    <parameter metaid="metaid_0000041" id="CIF" name="CIF (complex import factor)" value="5.672"
units="dimensionless" constant="false"/>
    <parameter metaid="metaid_0000042" id="konpS24nTIF1gamma" name="konpS24nTIF1gamma (Smad TIF1gamma
complex on-rate)" value="2e-03" units="pnMps"/>
    <parameter metaid="metaid_0000043" id="koffpS24nTIF1gamma" name="koffpS24nTIF1gamma (Smad
TIF1gamma complex off-rate)" value="1.6e-02" units="ps"/>
    <parameter metaid="metaid_0000044" id="koffpS2nTIF1gamma" name="koffpS2nTIF1gamma (Smad2 TIF1gamma
complex off-rate)" value="1.6e-02" units="ps"/>
    <parameter metaid="metaid_0000045" id="kinS4ub" name="kinS4ub (import rate for ubiquitinated
Smad4)" value="5.2e-03" units="ps"/>
    <parameter metaid="metaid_0000046" id="kdub" name="kdub (deubiquitination rate for S4ub)"
value="7e-03" units="pnMps"/>
  </listOfParameters>
  <listOfReactions>
  <reaction metaid="metaid_0000051" id="reaction_1" name="Ligand receptor complex formation"
reversible="false">
      <annotation>
        <rdf:RDF xmlns:rdf="http://www.w3.org/1999/02/22-rdf-syntax-ns#" xmlns:dc="http://purl.org/dc/
elements/1.1/" xmlns:dcterms="http://purl.org/dc/terms/" xmlns:vCard="http://www.w3.org/2001/vcard-
rdf/3.0#" xmlns:bqbiol="http://biomodels.net/biology-qualifiers/" xmlns:bqmodel="http://biomodels.net/
model-qualifiers/">
          <rdf:Description rdf:about="#metaid_0000051">
            <bqbiol:is>
              <rdf:Bag>
                <rdf:li rdf:resource="urn:miriam:obo.go:GO%3A0007181"/>
                <rdf:li rdf:resource="urn:miriam:obo.go:GO%3A0050431"/>
              </rdf:Bag>
            </bqbiol:is>
          </rdf:Description>
        </rdf:RDF>
      </annotation>
      <listOfReactants>
        <speciesReference species="RII"/>
        <speciesReference species="RI"/>
        <speciesReference species="TGFb"/>
      </listOfReactants>
      <listOfProducts>
        <speciesReference species="LR"/>
      </listOfProducts>
      <kineticLaw>
        <math xmlns="http://www.w3.org/1998/Math/MathML">
          <apply>
            <times/>
            <ci> ka </ci>
            <ci> TGFb </ci>
            <ci> RI </ci>
            <ci> RII </ci>
          </apply>
        </math>
      </kineticLaw>
```

```xml
    </reaction>
    <reaction metaid="metaid_0000052" id="reaction_2" name="Ligand receptor complex constitutive
degradation" reversible="false">
      <annotation>
        <rdf:RDF xmlns:rdf="http://www.w3.org/1999/02/22-rdf-syntax-ns#" xmlns:dc="http://purl.org/dc/
elements/1.1/" xmlns:dcterms="http://purl.org/dc/terms/" xmlns:vCard="http://www.w3.org/2001/vcard-
rdf/3.0#" xmlns:bqbiol="http://biomodels.net/biology-qualifiers/" xmlns:bqmodel="http://biomodels.net/
model-qualifiers/">
          <rdf:Description rdf:about="#metaid_0000052">
            <bqbiol:isVersionOf>
              <rdf:Bag>
                <rdf:li rdf:resource="urn:miriam:obo.go:GO%3A0030512"/>
              </rdf:Bag>
            </bqbiol:isVersionOf>
          </rdf:Description>
        </rdf:RDF>
      </annotation>
      <listOfReactants>
        <speciesReference species="LR"/>
      </listOfReactants>
      <kineticLaw>
        <math xmlns="http://www.w3.org/1998/Math/MathML">
          <apply>
            <times/>
            <ci> kcd </ci>
            <ci> LR </ci>
          </apply>
        </math>
      </kineticLaw>
    </reaction>
    <reaction metaid="metaid_0000053" id="reaction_3" name="Ligand independent complex degradation"
reversible="false">
      <annotation>
        <rdf:RDF xmlns:rdf="http://www.w3.org/1999/02/22-rdf-syntax-ns#" xmlns:dc="http://purl.org/dc/
elements/1.1/" xmlns:dcterms="http://purl.org/dc/terms/" xmlns:vCard="http://www.w3.org/2001/vcard-
rdf/3.0#" xmlns:bqbiol="http://biomodels.net/biology-qualifiers/" xmlns:bqmodel="http://biomodels.net/
model-qualifiers/">
          <rdf:Description rdf:about="#metaid_0000053">
            <bqbiol:isVersionOf>
              <rdf:Bag>
                <rdf:li rdf:resource="urn:miriam:obo.go:GO%3A0030512"/>
              </rdf:Bag>
            </bqbiol:isVersionOf>
          </rdf:Description>
        </rdf:RDF>
      </annotation>
      <listOfReactants>
        <speciesReference species="LR"/>
      </listOfReactants>
      <kineticLaw>
        <math xmlns="http://www.w3.org/1998/Math/MathML">
          <apply>
            <times/>
            <ci> klid </ci>
            <ci> LR </ci>
          </apply>
        </math>
      </kineticLaw>
    </reaction>
    <reaction metaid="metaid_0000054" id="reaction_4" name="Ligand receptor complex internalization"
reversible="false">
      <annotation>
        <rdf:RDF xmlns:rdf="http://www.w3.org/1999/02/22-rdf-syntax-ns#" xmlns:dc="http://purl.org/dc/
elements/1.1/" xmlns:dcterms="http://purl.org/dc/terms/" xmlns:vCard="http://www.w3.org/2001/vcard-
rdf/3.0#" xmlns:bqbiol="http://biomodels.net/biology-qualifiers/" xmlns:bqmodel="http://biomodels.net/
model-qualifiers/">
          <rdf:Description rdf:about="#metaid_0000054">
            <bqbiol:isVersionOf>
              <rdf:Bag>
                <rdf:li rdf:resource="urn:miriam:obo.go:GO%3A0030511"/>
              </rdf:Bag>
            </bqbiol:isVersionOf>
```

```
          </rdf:Description>
        </rdf:RDF>
      </annotation>
      <listOfReactants>
        <speciesReference species="LR"/>
      </listOfReactants>
      <listOfProducts>
        <speciesReference species="LRe"/>
      </listOfProducts>
      <kineticLaw>
        <math xmlns="http://www.w3.org/1998/Math/MathML">
          <apply>
            <times/>
            <ci> ki </ci>
            <ci> LR </ci>
          </apply>
        </math>
      </kineticLaw>
    </reaction>
    <reaction metaid="metaid_0000055" id="reaction_5" name="RI synthesis" reversible="false">
      <annotation>
        <rdf:RDF xmlns:rdf="http://www.w3.org/1999/02/22-rdf-syntax-ns#" xmlns:dc="http://purl.org/dc/
elements/1.1/" xmlns:dcterms="http://purl.org/dc/terms/" xmlns:vCard="http://www.w3.org/2001/vcard-
rdf/3.0#" xmlns:bqbiol="http://biomodels.net/biology-qualifiers/" xmlns:bqmodel="http://biomodels.net/
model-qualifiers/">
          <rdf:Description rdf:about="#metaid_0000055">
            <bqbiol:isVersionOf>
              <rdf:Bag>
                <rdf:li rdf:resource="urn:miriam:obo.go:GO%3A0006412"/>
              </rdf:Bag>
            </bqbiol:isVersionOf>
          </rdf:Description>
        </rdf:RDF>
      </annotation>
      <listOfProducts>
        <speciesReference species="RI"/>
      </listOfProducts>
      <kineticLaw>
        <math xmlns="http://www.w3.org/1998/Math/MathML">
          <ci> pRI </ci>
        </math>
      </kineticLaw>
    </reaction>
    <reaction metaid="metaid_0000056" id="reaction_6" name="RI constitutive degradation"
reversible="false">
      <annotation>
        <rdf:RDF xmlns:rdf="http://www.w3.org/1999/02/22-rdf-syntax-ns#" xmlns:dc="http://purl.org/dc/
elements/1.1/" xmlns:dcterms="http://purl.org/dc/terms/" xmlns:vCard="http://www.w3.org/2001/vcard-
rdf/3.0#" xmlns:bqbiol="http://biomodels.net/biology-qualifiers/" xmlns:bqmodel="http://biomodels.net/
model-qualifiers/">
          <rdf:Description rdf:about="#metaid_0000056">
            <bqbiol:isVersionOf>
              <rdf:Bag>
                <rdf:li rdf:resource="urn:miriam:obo.go:GO%3A0032801"/>
              </rdf:Bag>
            </bqbiol:isVersionOf>
          </rdf:Description>
        </rdf:RDF>
      </annotation>
      <listOfReactants>
        <speciesReference species="RI"/>
      </listOfReactants>
      <kineticLaw>
        <math xmlns="http://www.w3.org/1998/Math/MathML">
          <apply>
            <times/>
            <ci> kcd </ci>
            <ci> RI </ci>
          </apply>
        </math>
      </kineticLaw>
    </reaction>
```

```
    <reaction metaid="metaid_0000057" id="reaction_7" name="RI internalization" reversible="false">
      <annotation>
        <rdf:RDF xmlns:rdf="http://www.w3.org/1999/02/22-rdf-syntax-ns#" xmlns:dc="http://purl.org/dc/
elements/1.1/" xmlns:dcterms="http://purl.org/dc/terms/" xmlns:vCard="http://www.w3.org/2001/vcard-
rdf/3.0#" xmlns:bqbiol="http://biomodels.net/biology-qualifiers/" xmlns:bqmodel="http://biomodels.net/
model-qualifiers/">
          <rdf:Description rdf:about="#metaid_0000057">
            <bqbiol:isVersionOf>
              <rdf:Bag>
                <rdf:li rdf:resource="urn:miriam:obo.go:GO%3A0031623"/>
              </rdf:Bag>
            </bqbiol:isVersionOf>
          </rdf:Description>
        </rdf:RDF>
      </annotation>
      <listOfReactants>
        <speciesReference species="RI"/>
      </listOfReactants>
      <listOfProducts>
        <speciesReference species="RIe"/>
      </listOfProducts>
      <kineticLaw>
        <math xmlns="http://www.w3.org/1998/Math/MathML">
          <apply>
            <times/>
            <ci> ki </ci>
            <ci> RI </ci>
          </apply>
        </math>
      </kineticLaw>
    </reaction>
    <reaction metaid="metaid_0000058" id="reaction_8" name="RI recycling" reversible="false">
      <annotation>
        <rdf:RDF xmlns:rdf="http://www.w3.org/1999/02/22-rdf-syntax-ns#" xmlns:dc="http://purl.org/dc/
elements/1.1/" xmlns:dcterms="http://purl.org/dc/terms/" xmlns:vCard="http://www.w3.org/2001/vcard-
rdf/3.0#" xmlns:bqbiol="http://biomodels.net/biology-qualifiers/" xmlns:bqmodel="http://biomodels.net/
model-qualifiers/">
          <rdf:Description rdf:about="#metaid_0000058">
            <bqbiol:isVersionOf>
              <rdf:Bag>
                <rdf:li rdf:resource="urn:miriam:obo.go:GO%3A0001881"/>
              </rdf:Bag>
            </bqbiol:isVersionOf>
          </rdf:Description>
        </rdf:RDF>
      </annotation>
      <listOfReactants>
        <speciesReference species="RIe"/>
      </listOfReactants>
      <listOfProducts>
        <speciesReference species="RI"/>
      </listOfProducts>
      <kineticLaw>
        <math xmlns="http://www.w3.org/1998/Math/MathML">
          <apply>
            <times/>
            <ci> kr </ci>
            <ci> RIe </ci>
          </apply>
        </math>
      </kineticLaw>
    </reaction>
    <reaction metaid="metaid_0000059" id="reaction_9" name="Ligand Receptor complex recycling"
reversible="false">
      <annotation>
        <rdf:RDF xmlns:rdf="http://www.w3.org/1999/02/22-rdf-syntax-ns#" xmlns:dc="http://purl.org/dc/
elements/1.1/" xmlns:dcterms="http://purl.org/dc/terms/" xmlns:vCard="http://www.w3.org/2001/vcard-
rdf/3.0#" xmlns:bqbiol="http://biomodels.net/biology-qualifiers/" xmlns:bqmodel="http://biomodels.net/
model-qualifiers/">
          <rdf:Description rdf:about="#metaid_0000059">
            <bqbiol:isVersionOf>
              <rdf:Bag>
```

```
                <rdf:li rdf:resource="urn:miriam:obo.go:GO%3A0001881"/>
              </rdf:Bag>
            </bqbiol:isVersionOf>
          </rdf:Description>
        </rdf:RDF>
      </annotation>
      <listOfReactants>
        <speciesReference species="LRe"/>
      </listOfReactants>
      <listOfProducts>
        <speciesReference species="RI"/>
        <speciesReference species="RII"/>
      </listOfProducts>
      <kineticLaw>
        <math xmlns="http://www.w3.org/1998/Math/MathML">
          <apply>
            <times/>
            <ci> kr </ci>
            <ci> LRe </ci>
          </apply>
        </math>
      </kineticLaw>
    </reaction>
    <reaction metaid="metaid_0000060" id="reaction_10" name="RII synthesis" reversible="false">
      <annotation>
        <rdf:RDF xmlns:rdf="http://www.w3.org/1999/02/22-rdf-syntax-ns#" xmlns:dc="http://purl.org/dc/
elements/1.1/" xmlns:dcterms="http://purl.org/dc/terms/" xmlns:vCard="http://www.w3.org/2001/vcard-
rdf/3.0#" xmlns:bqbiol="http://biomodels.net/biology-qualifiers/" xmlns:bqmodel="http://biomodels.net/
model-qualifiers/">
          <rdf:Description rdf:about="#metaid_0000060">
            <bqbiol:isVersionOf>
              <rdf:Bag>
                <rdf:li rdf:resource="urn:miriam:obo.go:GO%3A0006412"/>
              </rdf:Bag>
            </bqbiol:isVersionOf>
          </rdf:Description>
        </rdf:RDF>
      </annotation>
      <listOfProducts>
        <speciesReference species="RII"/>
      </listOfProducts>
      <kineticLaw>
        <math xmlns="http://www.w3.org/1998/Math/MathML">
          <ci> pRII </ci>
        </math>
      </kineticLaw>
    </reaction>
    <reaction metaid="metaid_0000061" id="reaction_11" name="RII constitutive degradation"
reversible="false">
      <annotation>
        <rdf:RDF xmlns:rdf="http://www.w3.org/1999/02/22-rdf-syntax-ns#" xmlns:dc="http://purl.org/dc/
elements/1.1/" xmlns:dcterms="http://purl.org/dc/terms/" xmlns:vCard="http://www.w3.org/2001/vcard-
rdf/3.0#" xmlns:bqbiol="http://biomodels.net/biology-qualifiers/" xmlns:bqmodel="http://biomodels.net/
model-qualifiers/">
          <rdf:Description rdf:about="#metaid_0000061">
            <bqbiol:isVersionOf>
              <rdf:Bag>
                <rdf:li rdf:resource="urn:miriam:obo.go:GO%3A0032801"/>
              </rdf:Bag>
            </bqbiol:isVersionOf>
          </rdf:Description>
        </rdf:RDF>
      </annotation>
      <listOfReactants>
        <speciesReference species="RII"/>
      </listOfReactants>
      <kineticLaw>
        <math xmlns="http://www.w3.org/1998/Math/MathML">
          <apply>
            <times/>
            <ci> kcd </ci>
            <ci> RII </ci>
```

```
          </apply>
        </math>
      </kineticLaw>
    </reaction>
    <reaction metaid="metaid_0000062" id="reaction_12" name="RII internalization" reversible="false">
      <annotation>
        <rdf:RDF xmlns:rdf="http://www.w3.org/1999/02/22-rdf-syntax-ns#" xmlns:dc="http://purl.org/dc/
elements/1.1/" xmlns:dcterms="http://purl.org/dc/terms/" xmlns:vCard="http://www.w3.org/2001/vcard-
rdf/3.0#" xmlns:bqbiol="http://biomodels.net/biology-qualifiers/" xmlns:bqmodel="http://biomodels.net/
model-qualifiers/">
          <rdf:Description rdf:about="#metaid_0000062">
            <bqbiol:isVersionOf>
              <rdf:Bag>
                <rdf:li rdf:resource="urn:miriam:obo.go:GO%3A0031623"/>
              </rdf:Bag>
            </bqbiol:isVersionOf>
          </rdf:Description>
        </rdf:RDF>
      </annotation>
      <listOfReactants>
        <speciesReference species="RII"/>
      </listOfReactants>
      <listOfProducts>
        <speciesReference species="RIIe"/>
      </listOfProducts>
      <kineticLaw>
        <math xmlns="http://www.w3.org/1998/Math/MathML">
          <apply>
            <times/>
            <ci> ki </ci>
            <ci> RII </ci>
          </apply>
        </math>
      </kineticLaw>
    </reaction>
    <reaction metaid="metaid_0000063" id="reaction_13" name="RII recycling" reversible="false">
      <annotation>
        <rdf:RDF xmlns:rdf="http://www.w3.org/1999/02/22-rdf-syntax-ns#" xmlns:dc="http://purl.org/dc/
elements/1.1/" xmlns:dcterms="http://purl.org/dc/terms/" xmlns:vCard="http://www.w3.org/2001/vcard-
rdf/3.0#" xmlns:bqbiol="http://biomodels.net/biology-qualifiers/" xmlns:bqmodel="http://biomodels.net/
model-qualifiers/">
          <rdf:Description rdf:about="#metaid_0000063">
            <bqbiol:isVersionOf>
              <rdf:Bag>
                <rdf:li rdf:resource="urn:miriam:obo.go:GO%3A0001881"/>
              </rdf:Bag>
            </bqbiol:isVersionOf>
          </rdf:Description>
        </rdf:RDF>
      </annotation>
      <listOfReactants>
        <speciesReference species="RIIe"/>
      </listOfReactants>
      <listOfProducts>
        <speciesReference species="RII"/>
      </listOfProducts>
      <kineticLaw>
        <math xmlns="http://www.w3.org/1998/Math/MathML">
          <apply>
            <times/>
            <ci> kr </ci>
            <ci> RIIe </ci>
          </apply>
        </math>
      </kineticLaw>
    </reaction>
 <reaction metaid="metaid_0000064" id="reaction_14" name="Phosphorylation S2c" reversible="false">
      <annotation>
        <rdf:RDF xmlns:rdf="http://www.w3.org/1999/02/22-rdf-syntax-ns#" xmlns:dc="http://purl.org/dc/
elements/1.1/" xmlns:dcterms="http://purl.org/dc/terms/" xmlns:vCard="http://www.w3.org/2001/vcard-
rdf/3.0#" xmlns:bqbiol="http://biomodels.net/biology-qualifiers/" xmlns:bqmodel="http://biomodels.net/
model-qualifiers/">
```

```
          <rdf:Description rdf:about="#metaid_0000064">
            <bqbiol:isVersionOf>
              <rdf:Bag>
                <rdf:li rdf:resource="urn:miriam:ec-code:2.7.11.30"/>
                <rdf:li rdf:resource="urn:miriam:obo.go:GO%3A0004675"/>
              </rdf:Bag>
            </bqbiol:isVersionOf>
          </rdf:Description>
        </rdf:RDF>
      </annotation>
      <listOfReactants>
        <speciesReference species="LRe"/>
        <speciesReference species="S2c"/>
      </listOfReactants>
      <listOfProducts>
        <speciesReference species="LRe"/>
        <speciesReference species="pS2c"/>
      </listOfProducts>
      <kineticLaw>
        <math xmlns="http://www.w3.org/1998/Math/MathML">
          <apply>
            <times/>
            <ci> cytosol </ci>
            <ci> kphos </ci>
            <ci> LRe </ci>
            <ci> S2c </ci>
          </apply>
        </math>
      </kineticLaw>
    </reaction>
<reaction metaid="metaid_0000065" id="reaction_15" name="Formation pS24c">
      <annotation>
        <rdf:RDF xmlns:rdf="http://www.w3.org/1999/02/22-rdf-syntax-ns#" xmlns:dc="http://purl.org/dc/
elements/1.1/" xmlns:dcterms="http://purl.org/dc/terms/" xmlns:vCard="http://www.w3.org/2001/vcard-
rdf/3.0#" xmlns:bqbiol="http://biomodels.net/biology-qualifiers/" xmlns:bqmodel="http://biomodels.net/
model-qualifiers/">
          <rdf:Description rdf:about="#metaid_0000065">
            <bqbiol:isVersionOf>
              <rdf:Bag>
                <rdf:li rdf:resource="urn:miriam:obo.go:GO%3A0006461"/>
              </rdf:Bag>
            </bqbiol:isVersionOf>
          </rdf:Description>
        </rdf:RDF>
      </annotation>
      <listOfReactants>
        <speciesReference species="pS2c"/>
        <speciesReference species="S4c"/>
      </listOfReactants>
      <listOfProducts>
        <speciesReference species="pS24c"/>
      </listOfProducts>
      <kineticLaw>
        <math xmlns="http://www.w3.org/1998/Math/MathML">
          <apply>
            <times/>
                <ci> kon </ci>
                <ci> pS2c </ci>
                <ci> S4c </ci>
              </apply>
              <apply>
                <times/>
                <ci> koff </ci>
                <ci> pS24c </ci>
              </apply>
        </math>
      </kineticLaw>
    </reaction>
    <reaction metaid="metaid_0000066" id="reaction_16" name="Formation pS24n">
      <annotation>
        <rdf:RDF xmlns:rdf="http://www.w3.org/1999/02/22-rdf-syntax-ns#" xmlns:dc="http://purl.org/dc/
elements/1.1/" xmlns:dcterms="http://purl.org/dc/terms/" xmlns:vCard="http://www.w3.org/2001/vcard-
```

```xml
rdf/3.0#" xmlns:bqbiol="http://biomodels.net/biology-qualifiers/" xmlns:bqmodel="http://biomodels.net/
model-qualifiers/">
          <rdf:Description rdf:about="#metaid_0000066">
            <bqbiol:isVersionOf>
              <rdf:Bag>
                <rdf:li rdf:resource="urn:miriam:obo.go:GO%3A0006461"/>
              </rdf:Bag>
            </bqbiol:isVersionOf>
          </rdf:Description>
        </rdf:RDF>
      </annotation>
      <listOfReactants>
        <speciesReference species="pS2n"/>
        <speciesReference species="S4n"/>
      </listOfReactants>
      <listOfProducts>
        <speciesReference species="pS24n"/>
      </listOfProducts>
      <kineticLaw>
        <math xmlns="http://www.w3.org/1998/Math/MathML">
              <apply>
                <times/>
                <ci> kon </ci>
                <ci> pS2n </ci>
                <ci> S4n </ci>
              </apply>
              <apply>
                <times/>
                <ci> koff </ci>
                <ci> pS24n </ci>
              </apply>
        </math>
      </kineticLaw>
    </reaction>
   <reaction metaid="metaid_0000067" id="reaction_17" name="Formation pS22c">
      <annotation>
        <rdf:RDF xmlns:rdf="http://www.w3.org/1999/02/22-rdf-syntax-ns#" xmlns:dc="http://purl.org/dc/
elements/1.1/" xmlns:dcterms="http://purl.org/dc/terms/" xmlns:vCard="http://www.w3.org/2001/vcard-
rdf/3.0#" xmlns:bqbiol="http://biomodels.net/biology-qualifiers/" xmlns:bqmodel="http://biomodels.net/
model-qualifiers/">
          <rdf:Description rdf:about="#metaid_0000067">
            <bqbiol:isVersionOf>
              <rdf:Bag>
                <rdf:li rdf:resource="urn:miriam:obo.go:GO%3A0006461"/>
              </rdf:Bag>
            </bqbiol:isVersionOf>
          </rdf:Description>
        </rdf:RDF>
      </annotation>
      <listOfReactants>
        <speciesReference species="pS2c" stoichiometry="2"/>
      </listOfReactants>
      <listOfProducts>
        <speciesReference species="pS22c"/>
      </listOfProducts>
      <kineticLaw>
        <math xmlns="http://www.w3.org/1998/Math/MathML">
              <apply>
                <times/>
                <ci> kon </ci>
                <ci> pS2c </ci>
                <ci> pS2c </ci>
              </apply>
              <apply>
                <times/>
                <ci> koff </ci>
                <ci> pS22c </ci>
              </apply>
        </math>
      </kineticLaw>
    </reaction>
    <reaction metaid="metaid_0000068" id="reaction_18" name="Formation pS22n">
```

```xml
      <annotation>
        <rdf:RDF xmlns:rdf="http://www.w3.org/1999/02/22-rdf-syntax-ns#" xmlns:dc="http://purl.org/dc/
elements/1.1/" xmlns:dcterms="http://purl.org/dc/terms/" xmlns:vCard="http://www.w3.org/2001/vcard-
rdf/3.0#" xmlns:bqbiol="http://biomodels.net/biology-qualifiers/" xmlns:bqmodel="http://biomodels.net/
model-qualifiers/">
          <rdf:Description rdf:about="#metaid_0000068">
            <bqbiol:isVersionOf>
              <rdf:Bag>
                <rdf:li rdf:resource="urn:miriam:obo.go:GO%3A0006461"/>
              </rdf:Bag>
            </bqbiol:isVersionOf>
          </rdf:Description>
        </rdf:RDF>
      </annotation>
      <listOfReactants>
        <speciesReference species="pS2n" stoichiometry="2"/>
      </listOfReactants>
      <listOfProducts>
        <speciesReference species="pS22n"/>
      </listOfProducts>
      <kineticLaw>
        <math xmlns="http://www.w3.org/1998/Math/MathML">
              <apply>
                <times/>
                <ci> kon </ci>
                <ci> pS2n </ci>
                <ci> pS2n </ci>
              </apply>
              <apply>
                <times/>
                <ci> koff </ci>
                <ci> pS22n </ci>
              </apply>
        </math>
      </kineticLaw>
    </reaction>
  <reaction metaid="metaid_0000069" id="reaction_19" name="Shuttling S2">
      <annotation>
        <rdf:RDF xmlns:rdf="http://www.w3.org/1999/02/22-rdf-syntax-ns#" xmlns:dc="http://purl.org/dc/
elements/1.1/" xmlns:dcterms="http://purl.org/dc/terms/" xmlns:vCard="http://www.w3.org/2001/vcard-
rdf/3.0#" xmlns:bqbiol="http://biomodels.net/biology-qualifiers/" xmlns:bqmodel="http://biomodels.net/
model-qualifiers/">
          <rdf:Description rdf:about="#metaid_0000069">
            <bqbiol:isVersionOf>
              <rdf:Bag>
                <rdf:li rdf:resource="urn:miriam:obo.go:GO%3A0006913"/>
              </rdf:Bag>
            </bqbiol:isVersionOf>
          </rdf:Description>
        </rdf:RDF>
      </annotation>
      <listOfReactants>
        <speciesReference species="S2c"/>
      </listOfReactants>
      <listOfProducts>
        <speciesReference species="S2n"/>
      </listOfProducts>
      <kineticLaw>
        <math xmlns="http://www.w3.org/1998/Math/MathML">
          <apply>
            <minus/>
            <apply>
              <times/>
              <ci> kin </ci>
              <ci> S2c </ci>
            </apply>
            <apply>
              <times/>
              <ci> kex </ci>
              <ci> S2n </ci>
            </apply>
          </apply>
```

```
        </math>
      </kineticLaw>
    </reaction>
 <reaction metaid="metaid_0000070" id="reaction_20" name="Shuttling pS2">
      <annotation>
        <rdf:RDF xmlns:rdf="http://www.w3.org/1999/02/22-rdf-syntax-ns#" xmlns:dc="http://purl.org/dc/
elements/1.1/" xmlns:dcterms="http://purl.org/dc/terms/" xmlns:vCard="http://www.w3.org/2001/vcard-
rdf/3.0#" xmlns:bqbiol="http://biomodels.net/biology-qualifiers/" xmlns:bqmodel="http://biomodels.net/
model-qualifiers/">
          <rdf:Description rdf:about="#metaid_0000070">
            <bqbiol:isVersionOf>
              <rdf:Bag>
                <rdf:li rdf:resource="urn:miriam:obo.go:GO%3A0006913"/>
              </rdf:Bag>
            </bqbiol:isVersionOf>
          </rdf:Description>
        </rdf:RDF>
      </annotation>
      <listOfReactants>
        <speciesReference species="pS2c"/>
      </listOfReactants>
      <listOfProducts>
        <speciesReference species="pS2n"/>
      </listOfProducts>
      <kineticLaw>
        <math xmlns="http://www.w3.org/1998/Math/MathML">
          <apply>
            <minus/>
            <apply>
              <times/>
              <ci> kin </ci>
              <ci> pS2c </ci>
            </apply>
            <apply>
              <times/>
              <ci> kex </ci>
              <ci> pS2n </ci>
            </apply>
          </apply>
        </math>
      </kineticLaw>
    </reaction>
 <reaction metaid="metaid_0000071" id="reaction_21" name="Shuttling S4">
      <annotation>
        <rdf:RDF xmlns:rdf="http://www.w3.org/1999/02/22-rdf-syntax-ns#" xmlns:dc="http://purl.org/dc/
elements/1.1/" xmlns:dcterms="http://purl.org/dc/terms/" xmlns:vCard="http://www.w3.org/2001/vcard-
rdf/3.0#" xmlns:bqbiol="http://biomodels.net/biology-qualifiers/" xmlns:bqmodel="http://biomodels.net/
model-qualifiers/">
          <rdf:Description rdf:about="#metaid_0000071">
            <bqbiol:isVersionOf>
              <rdf:Bag>
                <rdf:li rdf:resource="urn:miriam:obo.go:GO%3A0006913"/>
              </rdf:Bag>
            </bqbiol:isVersionOf>
          </rdf:Description>
        </rdf:RDF>
      </annotation>
      <listOfReactants>
        <speciesReference species="S4c"/>
      </listOfReactants>
      <listOfProducts>
        <speciesReference species="S4n"/>
      </listOfProducts>
      <kineticLaw>
        <math xmlns="http://www.w3.org/1998/Math/MathML">
          <apply>
            <minus/>
            <apply>
              <times/>
              <ci> kin </ci>
              <ci> S4c </ci>
            </apply>
```

```xml
            <apply>
              <times/>
              <ci> kin </ci>
              <ci> S4n </ci>
            </apply>
          </apply>
        </math>
      </kineticLaw>
    </reaction>
    <reaction metaid="metaid_0000072" id="reaction_22" name="Import pS24" reversible="false">
      <annotation>
        <rdf:RDF xmlns:rdf="http://www.w3.org/1999/02/22-rdf-syntax-ns#" xmlns:dc="http://purl.org/dc/
elements/1.1/" xmlns:dcterms="http://purl.org/dc/terms/" xmlns:vCard="http://www.w3.org/2001/vcard-
rdf/3.0#" xmlns:bqbiol="http://biomodels.net/biology-qualifiers/" xmlns:bqmodel="http://biomodels.net/
model-qualifiers/">
          <rdf:Description rdf:about="#metaid_0000072">
            <bqbiol:isVersionOf>
              <rdf:Bag>
                <rdf:li rdf:resource="urn:miriam:obo.go:GO%3A0006913"/>
              </rdf:Bag>
            </bqbiol:isVersionOf>
          </rdf:Description>
        </rdf:RDF>
      </annotation>
      <listOfReactants>
        <speciesReference species="pS24c"/>
      </listOfReactants>
      <listOfProducts>
        <speciesReference species="pS24n"/>
      </listOfProducts>
      <kineticLaw>
        <math xmlns="http://www.w3.org/1998/Math/MathML">
          <apply>
            <times/>
            <ci> kin_CIF </ci>
            <ci> pS24c </ci>
          </apply>
        </math>
      </kineticLaw>
    </reaction>
    <reaction metaid="metaid_0000087" id="reaction_23" name="Import pS22" reversible="false">
      <annotation>
        <rdf:RDF xmlns:rdf="http://www.w3.org/1999/02/22-rdf-syntax-ns#" xmlns:dc="http://purl.org/dc/
elements/1.1/" xmlns:dcterms="http://purl.org/dc/terms/" xmlns:vCard="http://www.w3.org/2001/vcard-
rdf/3.0#" xmlns:bqbiol="http://biomodels.net/biology-qualifiers/" xmlns:bqmodel="http://biomodels.net/
model-qualifiers/">
          <rdf:Description rdf:about="#metaid_0000087">
            <bqbiol:isVersionOf>
              <rdf:Bag>
                <rdf:li rdf:resource="urn:miriam:obo.go:GO%3A0006913"/>
              </rdf:Bag>
            </bqbiol:isVersionOf>
          </rdf:Description>
        </rdf:RDF>
      </annotation>
      <listOfReactants>
        <speciesReference species="pS22c"/>
      </listOfReactants>
      <listOfProducts>
        <speciesReference species="pS22n"/>
      </listOfProducts>
      <kineticLaw>
        <math xmlns="http://www.w3.org/1998/Math/MathML">
          <apply>
            <times/>
            <ci> kin_CIF </ci>
            <ci> pS22c </ci>
          </apply>
        </math>
      </kineticLaw>
    </reaction>
    <reaction metaid="metaid_0000073" id="reaction_24" name="Dephos pS2n" reversible="false">
```

```
      <annotation>
        <rdf:RDF xmlns:rdf="http://www.w3.org/1999/02/22-rdf-syntax-ns#" xmlns:dc="http://purl.org/dc/
elements/1.1/" xmlns:dcterms="http://purl.org/dc/terms/" xmlns:vCard="http://www.w3.org/2001/vcard-
rdf/3.0#" xmlns:bqbiol="http://biomodels.net/biology-qualifiers/" xmlns:bqmodel="http://biomodels.net/
model-qualifiers/">
          <rdf:Description rdf:about="#metaid_0000073">
            <bqbiol:isVersionOf>
              <rdf:Bag>
                <rdf:li rdf:resource="urn:miriam:ec-code:3.1.3.16"/>
                <rdf:li rdf:resource="urn:miriam:obo.go:GO%3A0004722"/>
              </rdf:Bag>
            </bqbiol:isVersionOf>
          </rdf:Description>
        </rdf:RDF>
      </annotation>
      <listOfReactants>
        <speciesReference species="pS2n"/>
        <speciesReference species="PPase"/>
      </listOfReactants>
      <listOfProducts>
        <speciesReference species="S2n"/>
        <speciesReference species="PPase"/>
      </listOfProducts>
      <kineticLaw>
        <math xmlns="http://www.w3.org/1998/Math/MathML">
          <apply>
            <times/>
            <ci> kdephos </ci>
            <ci> pS2n </ci>
            <ci> PPase </ci>
          </apply>
        </math>
      </kineticLaw>
    </reaction>
<reaction metaid="metaid_0000074" id="reaction_25" name="Formation pS24nTIF1gamma" reversible="false">
      <annotation>
        <rdf:RDF xmlns:rdf="http://www.w3.org/1999/02/22-rdf-syntax-ns#" xmlns:dc="http://purl.org/dc/
elements/1.1/" xmlns:dcterms="http://purl.org/dc/terms/" xmlns:vCard="http://www.w3.org/2001/vcard-
rdf/3.0#" xmlns:bqbiol="http://biomodels.net/biology-qualifiers/" xmlns:bqmodel="http://biomodels.net/
model-qualifiers/">
        </rdf:RDF>
      </annotation>
      <listOfReactants>
        <speciesReference species="pS2n"/>
        <speciesReference species="TIF1gamma"/>
      </listOfReactants>
      <listOfProducts>
        <speciesReference species="pS24nTIF1gamma"/>
      </listOfProducts>
      <kineticLaw>
        <math xmlns="http://www.w3.org/1998/Math/MathML">
          <apply>
            <times/>
            <ci> konpS24nTIF1gamma </ci>
            <ci> pS2n </ci>
            <ci> TIF1gamma </ci>
          </apply>
        </math>
      </kineticLaw>
    </reaction>
<reaction metaid="metaid_0000075" id="reaction_26" name="Dissociation pS24nTIF1gamma"
reversible="false">
      <annotation>
        <rdf:RDF xmlns:rdf="http://www.w3.org/1999/02/22-rdf-syntax-ns#" xmlns:dc="http://purl.org/dc/
elements/1.1/" xmlns:dcterms="http://purl.org/dc/terms/" xmlns:vCard="http://www.w3.org/2001/vcard-
rdf/3.0#" xmlns:bqbiol="http://biomodels.net/biology-qualifiers/" xmlns:bqmodel="http://biomodels.net/
model-qualifiers/">
        </rdf:RDF>
      </annotation>
      <listOfReactants>
        <speciesReference species="pS24nTIF1gamma"/>
      </listOfReactants>
```

```
      <listOfProducts>
        <speciesReference species="pS2nTIF1gamma"/>
        <speciesReference species="S4ub_n"/>
      </listOfProducts>
      <kineticLaw>
        <math xmlns="http://www.w3.org/1998/Math/MathML">
          <apply>
            <times/>
            <ci> koffpS24nTIF1gamma </ci>
            <ci> pS24nTIF1gamma </ci>
          </apply>
        </math>
      </kineticLaw>
    </reaction>
<reaction metaid="metaid_0000076" id="reaction_27" name="Dissociation pS2nTIF1gamma"
reversible="false">
      <annotation>
        <rdf:RDF xmlns:rdf="http://www.w3.org/1999/02/22-rdf-syntax-ns#" xmlns:dc="http://purl.org/dc/
elements/1.1/" xmlns:dcterms="http://purl.org/dc/terms/" xmlns:vCard="http://www.w3.org/2001/vcard-
rdf/3.0#" xmlns:bqbiol="http://biomodels.net/biology-qualifiers/" xmlns:bqmodel="http://biomodels.net/
model-qualifiers/">
        </rdf:RDF>
      </annotation>
      <listOfReactants>
        <speciesReference species="pS2nTIF1gamma"/>
      </listOfReactants>
      <listOfProducts>
        <speciesReference species="pS2n"/>
        <speciesReference species="TIF1gamma"/>
      </listOfProducts>
      <kineticLaw>
        <math xmlns="http://www.w3.org/1998/Math/MathML">
          <apply>
            <times/>
            <ci> koffpS2nTIF1gamma </ci>
            <ci> pS2nTIF1gamma </ci>
          </apply>
        </math>
      </kineticLaw>
    </reaction>
<reaction metaid="metaid_0000077" id="reaction_28" name="transport S4ub_n" reversible="false">
      <annotation>
        <rdf:RDF xmlns:rdf="http://www.w3.org/1999/02/22-rdf-syntax-ns#" xmlns:dc="http://purl.org/dc/
elements/1.1/" xmlns:dcterms="http://purl.org/dc/terms/" xmlns:vCard="http://www.w3.org/2001/vcard-
rdf/3.0#" xmlns:bqbiol="http://biomodels.net/biology-qualifiers/" xmlns:bqmodel="http://biomodels.net/
model-qualifiers/">
        </rdf:RDF>
      </annotation>
      <listOfReactants>
        <speciesReference species="S4ub_n"/>
      </listOfReactants>
      <listOfProducts>
        <speciesReference species="S4ub_c"/>
      </listOfProducts>
      <kineticLaw>
        <math xmlns="http://www.w3.org/1998/Math/MathML">
          <apply>
            <times/>
            <ci> kinS4ub </ci>
            <ci> S4ub_n </ci>
          </apply>
        </math>
      </kineticLaw>
    </reaction>
<reaction metaid="metaid_0000078" id="reaction_29" name="deubiquitination S4ub_c" reversible="false">
      <annotation>
        <rdf:RDF xmlns:rdf="http://www.w3.org/1999/02/22-rdf-syntax-ns#" xmlns:dc="http://purl.org/dc/
elements/1.1/" xmlns:dcterms="http://purl.org/dc/terms/" xmlns:vCard="http://www.w3.org/2001/vcard-
rdf/3.0#" xmlns:bqbiol="http://biomodels.net/biology-qualifiers/" xmlns:bqmodel="http://biomodels.net/
model-qualifiers/">
        </rdf:RDF>
      </annotation>
```

```
      <listOfReactants>
        <speciesReference species="S4ub_c"/>
        <speciesReference species="FAM"/>
      </listOfReactants>
      <listOfProducts>
        <speciesReference species="S4c"/>
        <speciesReference species="FAM"/>
      </listOfProducts>
      <kineticLaw>
        <math xmlns="http://www.w3.org/1998/Math/MathML">
          <apply>
            <times/>
            <ci> FAM </ci>
            <ci> S4ub_c </ci>
          </apply>
        </math>
      </kineticLaw>
    </reaction>
  </listOfReactions>
</model>
</sbml>
```
